# Tubular Mas receptor mediates lipid-induced kidney injury

**DOI:** 10.1038/s41419-020-03375-z

**Published:** 2021-01-21

**Authors:** Yonglun Kong, Xiaoduo Zhao, Miaojuan Qiu, Yu Lin, Pinning Feng, Suchun Li, Baien Liang, Qing Zhu, Hui Huang, Chunling Li, Weidong Wang

**Affiliations:** 1 Department of Pathophysiology, Zhongshan School of Medicine, Sun Yat-sen University, Guangzhou, 510080 China; 2 Research Center, The Seventh Affliated Hospital, Sun Yat-sen University, Shenzhen, 518107 China; 3 Department of Pathology, Zhujiang Hospitial, Southern Medical University, Guangzhou, 510282 China; 4 Department of Clinical Laboratory, The First Affiliated Hospital, Sun Yat-sen University, Guangzhou, China; 5 Guangdong Metabolic Disease Research Center of Integrated Chinese and Western Medicine, Guangdong Pharmaceutical University, Guangzhou, 510006 China; 6 Department of Cardiology, The Eighth Affiliated Hospital, Sun Yat-sen University, Shenzhen, 518033 China; 7 Institute of Hypertension, Zhongshan School of Medicine, Sun Yat-sen University, Guangzhou, 510080 China; 8 Department of Nephrology, The Seventh Affiliated Hospital, Sun Yat-sen University, Shenzhen, China

**Keywords:** Autophagy, Chronic kidney disease

## Abstract

Obesity-related kidney diseases are becoming serious health problems worldwide, yet the mechanism by which obesity causes kidney injury is not fully understood. The purpose of current study was to investigate the role of Mas receptor in lipid-induced kidney injury. In mice fed with high-fat diet (HFD), the protein abundance of markers of autophagy, endoplasmic reticulum stress (ER stress) and apoptosis was dramatically increased in the kidney cortex, which was markedly prevented by Mas deletion (Mas^−/−^) or Mas receptor antagonist A779. Palmitic acid (PA) induced persistently increased autophagy, ER stress, and apoptosis as well as mitochondrial injuries in primary cultured proximal tubular cells from wild type, but not from Mas^−/−^ mice. In human proximal tubular HK2 cells, PA-induced autophagy and ER stress was aggravated by Mas agonists Ang (1–7) or AVE0991, but attenuated by A779 or Mas knockdown. Stimulation of Mas resulted in elevated intracellular calcium levels [Ca^2+^]_i_ in HK2 cells treated with PA, whereas inhibition or knockdown of Mas decreased [Ca^2+^]_i_. Mitochondrial outer membrane located voltage-dependent anion channel (VDAC1) was markedly upregulated in HK2 cells treated with PA, which was associated with impaired mitochondrial morphology and depolarization. These were enhanced by AVE0991 and suppressed by A779 or Mas knockdown. Mas knockdown in HK2 cells prevented impaired interactions among VDAC1, autophagy adaptor P62, and ubiquitin, induced by PA, leading to a potential ubiquitination of VDAC1. In conclusion, Mas receptor-mediated lipid-induced impaired autophagy and ER stress in the kidney, likely contributing to tubular injuries in obesity-related kidney diseases.

## Introduction

Obesity-related kidney diseases are becoming serious health problems worldwide, yet the mechanism by which obesity causes kidney injury is not fully understood. Accumulation of saturated free fatty acid and their metabolites within renal epithelial cells produces lipotoxicity, resulting in significant cellular dysfunction and injuries. Although proteinuria and declining GFR was well documented in renal lipotoxicity, more subtle manifestations of lipid-induced renal tubular injury are in need to further investigate. Lipid accumulation causes tubular cell damage through various mechanisms, including the inflammatory response, reactive oxygen specis (ROS) production, mitochondrial dysfunction, endoplasmic reticulum stress (ER stress), autophagy deregulation, and apoptosis^1,2^.

It is well known that renin-angiotensin system (RAS) plays a crucial role in obesity-related kidney diseases. Each RAS component has been identified in the kidney, suggesting the role of the local/tissue and intracellular RAS in the development and progression of renal diseases. We have recently demonstrated that blockade of RAS with direct renin inhibitor aliskiren, angiotensin-II type 1 receptor blocker valsartan, or chymase inhibitor chymostatin effectively attenuated tubular epithelial cell injuries induced by saturated fatty acid palmitic acid (PA) or high-fat diet (HFD) in mice^3,4^. As an alternative pathway of the RAS, angiotensin 1–7 (Ang (1–7)) and its G-protein coupled receptor Mas^5,6^ (encoded by Mas1 gene, also named as Mrgprs^7^, the term “Mas” was used in the present article) has been extensively examined. Angiotensin converting enzyme 2 (ACE2) is one of the main enzymes involved in the production of Ang (1–7) from angiotensin-II. Potential downstream signaling pathways of Mas stimulated by Ang (1–7) and related analogs include the phospholipase A2 and the phosphoinositide 3-kinase/AKT pathway, as well as the phospholipase C and Ca^2+^ signaling pathway^8^. Recent studies demonstrated that Ang (1–7) increased intracellular calcium levels in microperfused proximal tubular cells, which was markedly inhibited by A779^9,10^, an antagonist of Mas. In general, ACE2-Ang (1–7)-Mas receptor axis counteracts the classical RAS (e.g., angiotensin-II), resulting in vasodilation, anti-inflammation, anti-fibrosis, and anti-apoptosis, conferring beneficial effects in the settings of cardiovascular diseases^11,12^. However, in the kidney, although the majority of studies describes ACE2-Ang (1–7)-Mas receptor axis as a protective factor in different kidney diseases, the complexity and controversial actions of Ang (1–7)/Mas has been recognized recently^11^. Genetic deletion of the Mas led to glomerular hyperfiltration and microalbuminuria^13^, Ang (1–7) was found fail to improve albuminuria in 5/6 nephroectomized rats^14^. A recent study demonstrated that Mas deficiency diminished renal damage in models of renal insufficiency as unilateral ureteral obstruction and ischemia/reperfusion injury, while the infusion of Ang (1–7) to wild type mice aggravated the inflammatory response^15^. The role of Ang (1–7)/Mas receptor signaling pathway in obesity-related kidney diseases also remains clarified. Mas deficiency was shown to lead to metabolic abnormalities, e.g., dyslipidemia and type 2 diabetes in FVB/N mice^16^, but in another studies, diet-induced obesity was less pronounced in Mas-knockout FVB/N than in wild type mice^17^, and Mas deficiency in C57BL/6 mice have no effect on lipid metabolism^18^.

Autophagy is a highly regulated lysosomal protein degradation pathway, which maintains intracellular homeostasis and cell integrity by removing damaged organelles, protein aggregates, other macromolecules, and recycling some metabolites^19^. As a pro-survival mechanism, autophagy is elevated in response to various cellular stresses, e.g., ER stress. Various stresses, for example, lipid overload, cause accumulation of unfolded and/or misfolded proteins in the ER, inducing unfolded protein response (UPR) pathway and ER stress, which is supposed to attenuate protein translation, promote expression of chaperones and degradation of unfolded proteins. Unresolved and prolonged stress causes apoptosis and programmed cell death^20^.

Dysfunction of the ER together with ER stress and autophagy contributes to the development and progression of kidney disease^21,22^. A great number of evidence has demonstrated that ER stress evokes autophagy^19^, but whether autophagy induces ER stress in some pathophysiological conditions is not fully understood. PA, the most abundant circulating fatty acid in vivo^3^, induces both ER stress^4^ and autophagy in proximal tubular cells (PTCs)^23,24^. Currently, there is a few data clearly deciphered interaction between ER stress and autophagy in the kidney. More works are needed to integrate the two signaling pathways responsible for the induction of ER stress upon autophagy and the cellular consequences in kidney, in particular, lipid-induced tubular injuries, as free fatty acids (FFAs) is a major source of renal ATP synthesis, particularly in PTCs. In the progression of obesity or diabetes, PTCs consistently uptake FAs from circulation, lipid metabolism in PTCs are becoming insufficient when compared with the liver and adipose tissue, which have better lipid handling abilities.

Optimum Ca^2+^ movement across the cytoplasma membrane and ER membrane ensures the proper function of intracellular calcium-dependent kinases and proteases. Indeed, perturbation of this dynamic intracellular Ca^2+^ levels can lead to activation of Ca^2+^-regulated pathways, including autophagy, mitochondrial and ER stress. Ca^2+^ transport across the outer membrane of mitochondria to the inter-membranal space is mediated by voltage-dependent anion channel, VDAC^25–27^. VDAC1 is now recognized as a key player in mitochondria-mediated apoptosis. VDAC inhibition prevented mitochondria membrane potential dissipation and apoptosis^28,29^ and increased VDAC level is responsible for increased mitochondrial Ca^2+^ concentration, ROS production, and mPTP opening activity^30^, which are known to induce apoptosis. PA significantly increased mitochondrial ROS and decreased mitochondrial membrane potential in autophagy-deficient PTCs^23^, suggesting an association between autophagy and mitochondrial function in response to PA overload, however, a role of Ca^2+^-VDAC1 pathway in mitochondria dysfunctions associated with impaired autophagy in PTCs is unknown.

In the current study, we aim to investigate whether Mas signaling activation is involved in lipid-induced PTCs injury and the potential mechanism in vivo and in vitro.

## Results

### Mas deletion prevented high-fat diet-induced kidney injury

Mas^+/+^ mice with HFD exhibited an about 60% increase in body weight and elevated serum glucose, FFA and cholesterol levels. Remarkably increased urinary albumin and urine albumin to creatinine ratio (UACR) was also found when compared to mice with normal chow (Table 1). Body weight of Mas^−/−^ mice showed a mild, but significant increase compared to wild type mice (Table 1). There was no significant differences in serum glucose and lipid profile between Mas^+/+^ and Mas^−/−^ mice with normal chow, but urinary albumin and uACR was mildly increased in Mas^−/−^ mice (Table 1). In Mas^−/−^ mice with HFD, body weight increased 20%, but serum glucose and FFA levels are not different from Mas^−/−^ mice with normal chow. Urinary albumin was also increased, but not as high as seen in Mas^+/+^ mice with HFD (Table 1). These data showed Mas deletion likely affected body metabolism and renal filtration.

**Table 1:** Functional data of Mas^+/+^ and Mas^−/−^ mice with or without HFD.

Groups	WT-CTL (*n* = 6)	WT-HFD (*n* = 6)	KO-CTL (*n* = 6)	KO-HFD (*n* = 6)
Body weight (g)	28.7 ± 1.9	46.1 ± 2.7*	31.7 ± 0.5*	37.7 ± 1.4^#&^
KW/TL (g/cm)	0.13 ± 0.007	0.14 ± 0.009	0.14 ± 0.005	0.14 ± 0.009
S-Glu (mmol/dL)	4.20 ± 0.82	9.46 ± 0.63*	5.35 ± 0.19	5.76 ± 0.58^&^
S-FFA (mmol/L)	699 ± 65	865 ± 54*	718 ± 73	599 ± 31^&^
S-Chol (mg/dL)	1.86 ± 0.22	3.38 ± 0.37*	1.66 ± 0.07	2.55 ± 0.26^#&^
S-TG (mg/dL)	0.56 ± 0.09	0.72 ± 0.04	0.50 ± 0.06	0.60 ± 0.07
S-HDL (mmol/L)	1.31 ± 0.15	2.43 ± 0.30*	1.20 ± 0.04	1.81 ± 0.22^&^
S-LDL (mmol/L)	0.50 ± 0.06	0.88 ± 0.06*	0.46 ± 0.03	0.76 ± 0.07*^#^
S-Cr (μmol/L)	9.47 ± 0.72	9.38 ± 0.62	11.1 ± 1.79	11.3 ± 0.73
C_Cr_ (μl/min/g)	11.40 ± 1.83	5.33 ± 1.00*	12.92 ± 3.70	11.43 ± 1.32
U-ALB (μg/24 h)	1.86 ± 1.05	19.00 ± 4.38*	3.17 ± 0.43	7.38 ± 1.37^#&^
UACR	0.05 ± 0.01	0.55 ± 0.12*	0.09 ± 0.02*	0.11 ± 0.02^&^

*HFD* high-fat diet, *KW* kidney weight, *TL* tibia length, *S-Glu* serum glucose, *S-FFA* serum-free fatty acid, *S-Chol* serum cholesterol, *S-TG* serum triglycerides, *S-HDL* serum high-density lipoprotein, *S-LDL* serum low-density lipoprotein, *S-Cr* serum creatinine, *Ccr* clearance of creatinine, *U-Alb* urine albumin, *UACR* urine albumin to creatinine ratio.

*P < 0.05 when compared with WT-CTL group, ^#^*P* < 0.05 when compared with KO-CTL group, ^&^*P* < 0.05 compared with WT-HFD.

**Fig. 1:**
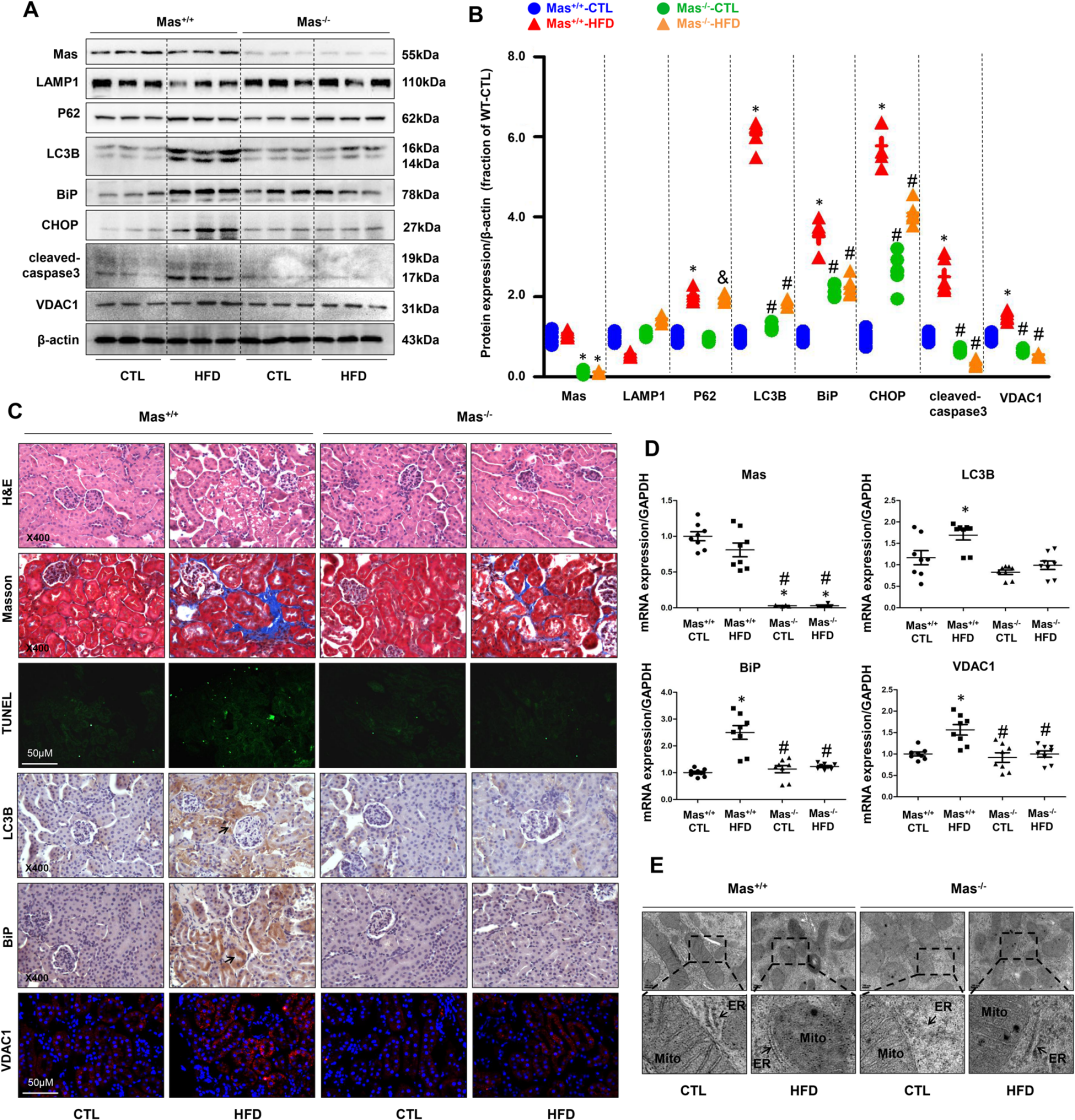
Mas receptor deletion prevented high-fat diet-induced autophagy, ER stress, and apopotosis in the cortex of kidneys in mice. **A** and **B** Representative immunoblots and corresponding densitometry analysis of Mas, LAMP1, P62, LC3B, BiP, CHOP, cleaved-caspase-3 and VDAC1 protein abundance in the kidney cortex of Mas^+/+^ CTL, Mas^+/+^ HFD, Mas^−/−^ CTL and Mas^−/−^ HFD mice. **C** Representative photomicrographs of H&E, Masson staining and TUNEL staining in the kidney of Mas^+/+^ CTL, Mas^+/+^ HFD, Mas^−/−^ CTL and Mas^−/−^ HFD mice. Immunohistochemistry and immunofluorescence of LC3B, BiP, and VDAC1 in the kidney of Mas^+/+^ CTL, Mas^+/+^ HFD, Mas^−/−^ CTL and Mas^−/−^ HFD mice. **D** mRNA levels of VDAC1, LC3B and BiP in the kidney cortex of Mas^+/+^ CTL, Mas^+/+^ HFD, Mas^−/−^ CTL and Mas^−/−^ HFD mice. **E** Representative images of mitochondria and endoplasmic reticulum by transmission electron microscopy (TEM) in proximal tubular cells prepared from Mas^+/+^ CTL, Mas^+/+^ HFD, Mas^−/−^ CTL and Mas^−/−^ HFD mice. CTL controls, HFD high-fat diet. Data are shown as mean ± SEM; *P < 0.05 compared with Mas^+/+^ CTL; ^#^*P* < 0.05 compared with Mas^+/+^ HFD.

Next we examined expressions of autophagy and ER stress markers in the kidneys of mice with or without HFD. In Mas^+/+^ mice with HFD, the protein abundance of LC3B and P62, two markers of autophagy, was markedly upregulated in the renal cortex compared to Mas^+/+^ mice with normal chow, in particular, LC3B expression showed an about 6-fold increase, whereas LAMP1 expression was clearly decreased, indicating an induction of autophagy (Fig. 1A, B). In contrast, in Mas^−/−^ mice with HFD, LC3B protein expression was only increased to 2-folds (Fig. 1A, B). There were no significant differences in protein expression of LC3B, P62, and LAMP1 between Mas^+/+^ and Mas^−/−^ mice with normal chow (Fig. 1A, B). The abundance of BiP (also called GRP78), a marker of ER stress, was dramatically increased in the kidney of Mas^+/+^ mice with HFD, BiP expression was also mildly upregulated in Mas^−/−^ mice with or without HFD compared to Mas^+/+^ mice with normal chow (Fig. 1A, B). The protein expression of CHOP, another marker of ER stress, and cleaved-caspase-3, a marker of apoptosis, were also dramatically increased in Mas^+/+^ mice with HFD, but not in Mas^−/−^ mice with or without HFD (Fig. 1A, B). Similarly, VDAC1 protein abundance was increased in Mas^+/+^ mice with HFD, but not in Mas^−/−^ mice with or without HFD (Fig. 1A, B).

H&E and Masson’s staining showed more proximal tubular injuries and fibrosis in kidney cortex of Mas^+/+^ mice fed with HFD than that in Mas^−/−^ mice. Few difference in renal histology was seen between Mas^+/+^ and Mas^−/−^ mice fed with normal chow (Fig. 1C). TUNEL assay showed marked cell death in renal cortex of Mas^+/+^ mice fed with HFD compared with Mas^−/−^ mice (Fig. 1C). Immunohistochemistry and immunofluorescence demonstrated increased staining intenstiy of LC3B, BiP and VDAC1 proteins in the proximal tubular cells of Mas^+/+^ mice with HFD, whereas in Mas^−/−^ mice with HFD, lableling density of these proteins was not as strong as seen in Mas^+/+^ mice with HFD, although it might be stronger than that in Mas^−/−^ mice with normal chow (Fig. 1C). Consistent with increased protein expression, mRNA levels of LC3B, BiP and VDAC1 were remarkably upregulated in renal cortex of Mas^+/+^ mice with HFD, while in Mas^−/−^ mice with HFD, these mRNA levels were not changed when compared with Mas^−/−^ mice with normal chow (Fig. 1D). Transmission electronic microscopy (TEM) images revealed more swollen mitochondria with disorganized and fragmented cristae in the proximal tubular cells of the kidney in Mas^+/+^ mice with HFD than those in Mas^−/−^ mice with HFD (Fig. 1E). DRP1 (mitochondrial dynamin-related protein 1) protein abundance (supplementary data II Fig. S1A, B) and mRNA levels (Fig. S1C) were significantly increased in the renal cortex of mice fed with HFD, which was markedly prevented by Mas deletion. These data likely suggested that Mas might be involved in HFD-associated tubular cell injuries in mice.

Renal proximal tubular cells from Mas^+/+^ and Mas^−/−^ mice were prepared as previously reported^31^. PA treatment caused persistently increased protein abundance of LC3B and VDAC1 in PTCs prepared from Mas^+/+^ but not Mas^−/−^ mice (Supplementary data II Fig. S2A, B). BiP protein expression was dramatically upregulated at 12 h and 24 h after PA treatment, which was associated with increased expression of cleaved-caspase-3 in PTCs of Mas^+/+^ mice (Fig. S2A, B). In contrast, with PA treatment protein abundance of these two was generally maintained in PTCs of Mas^−/−^ mice (Fig. S2A, B), although cleaved-caspase-3 was showed a slight increase at the 24 h, indicating an involvement of Mas in mediating PA-induced cell injury. Mito-tracker label assay showed improved mitochondrial morphology in PTCs prepared from Mas^−/−^ mice than that from Mas^+/+^ mice after PA treatment (Fig. S2C).

### Mas antagonist A779 prevented high-fat diet-induced kidney injury

**Fig. 2:**
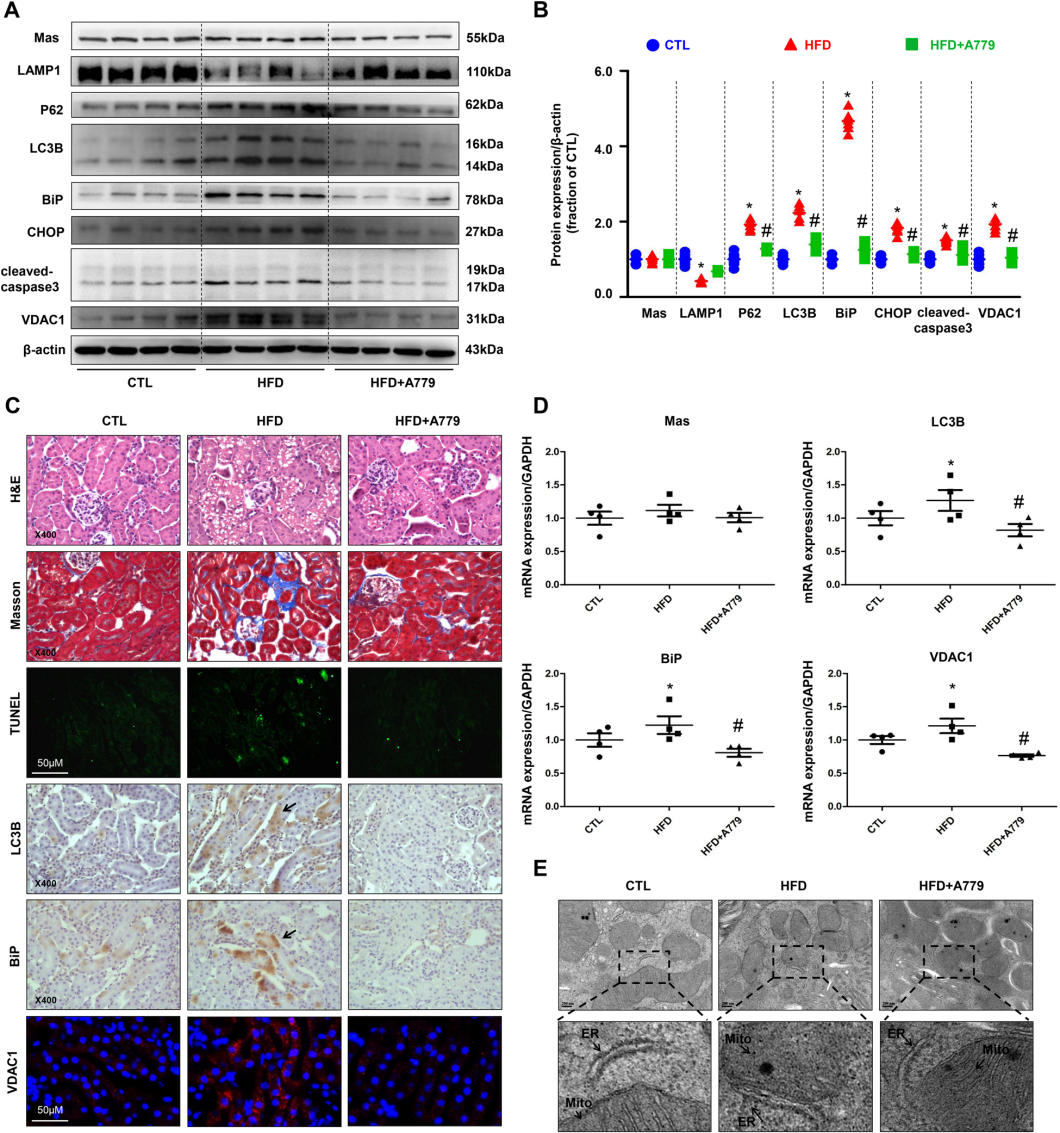
Mas antagonist A779 prevented high-fat diet-induced autophagy, ER stress, and apoptosis in the cortex of kidneys in mice. **A** and **B** Representative immunoblots and corresponding densitometry analysis of Mas, LAMP1, P62, LC3B, BiP, CHOP, cleaved-caspase-3 and VDAC1 protein abundance in the kidney cortex of CTL, HFD, and HFD+A779 mice. **C** Representative photomicrographs of H&E, Masson staining and TUNEL staining the kidney of CTL, HFD, and HFD+A779 mice. Immunohistochemistry and immunofluorescence of LC3B, BiP, and VDAC1 in the kidney of CTL, HFD, and HFD+A779 mice. **D** mRNA levels of Mas, LC3B, BiP and VDAC1 in the kidney cortex of CTL, HFD, and HFD+A779 mice. **E** Representative images of mitochondria and endoplasmic reticulum by transmission electron microscopy (TEM) in proximal tubular cells prepared from HFD mice with or without A779 treatment. CTL, controls; HFD, high-fat diet; HFD+A779, high-fat diet plus A779 treatment. Data are shown as mean ± SEM; *P < 0.05 compared with CTL; ^#^*P* < 0.05 compared with HFD.

To further examine the role of Mas in the kidney, Mas receptor antagonist A779 was used in C57BL/6 mice fed HFD in combination with unilateral nephrectomy, to accelerate the progression of renal injury^32^. Mice with HFD exhibited elevated serum glucose, serum FFA, urine albumin, and UACR compared to mice with normal chow (Table 2). Notable decreases in the serum glucose and FFA levels were observed in HFD mice with A779 treatment. Reductions in urine albumin and UACR was also found in HFD mice treated with A779 (Table 2). Western blot revealed dramatically increased abundance of LC3B, P62, BiP, CHOP, and VDAC1 proteins in renal cortex of HFD mice, which were at least partially prevented by A779 treatment (Fig. 2A, B). A779 treatment prevented tubular injury, fibrosis and tubular cell death (Fig. 2C). Immunohistochemistry and immunofluorescence confirmed increased protein expression of LC3B, BiP and VDAC1 in the proximal tubular cells of HFD mice, which was obviously decreased after A779 treatment (Fig. 2C). mRNA expression levels of LC3B, BiP, and VDAC1 were also increased in HFD mice, which was notably reduced by A779 (Fig. 2D). TEM revealed more mitochondrial injury in the proximal tubular cells of the kidney in HFD mice than those in control mice, which was at least partially ameliorated by A779 (Fig. 2E). Consistent with alterations in mitochondrial morphology, DRP1 protein abundance and mRNA levels were dramtically increased in the renal cortex of mice fed with HFD, which was markedly prevented by A779 (supplementary data II, Fig. S3A–C). These results suggest that A779 may partially prevent proximal tubular cell injuries induced by high-fat diet in mice.

**Table 2:** Functional data of HFD mice with or without A779 treatment.

Groups	CTL (*n* = 6)	HFD (*n* = 6)	HFD + A779 (*n* = 6)
Body weight (g)	24.6 ± 1.3	33.1 ± 2.5*	28.8 ± 1.2*^#^
KW/TL (g/cm)	0.13 ± 0.01	0.17 ± 0.01*	0.17 ± 0.01*
S-Glu (mmol/dL)	4.55 ± 0.52	12.37 ± 0.82*	8.64 ± 0.01*^#^
S-FFA (mmol/L)	593 ± 35	992 ± 75*	695 ± 83*^#^
S-Chol (mg/dL)	2.67 ± 0.06	3.47 ± 0.23*	3.15 ± 0.24*
S-TG (mg/dL)	0.51 ± 0.06	0.53 ± 0.04	0.59 ± 0.15
S-HDL (mmol/L)	2.06 ± 0.02	2.16 ± 0.05*	2.31 ± 0.02*^#^
S-LDL (mmol/L)	0.56 ± 0.02	0.61 ± 0.02*	0.82 ± 0.05*^#^
S-Cr (μmol/L)	12.2 ± 0.91	12.8 ± 0.49	14.3 ± 0.59
C_Cr_ (μl/min/g)	11.65 ± 1.44	5.60 ± 0.67*	7.37 ± 0.49*
U-Alb (μg/24 h)	4.22 ± 1.05	13.17 ± 3.25*	6.78 ± 1.89^#^
UACR	0.12 ± 0.02	0.21 ± 0.01*	0.17 ± 0.01*^#^

*HFD* high-fat diet, *KW* kidney weight, *TL* tibia length, *S-Glu* serum glucose, *S-FFA* serum-free fatty acid, *S-Chol* serum cholesterol, *S-TG* serum triglycerides, *S-HDL* serum high-density lipoprotein, *S-LDL* serum low-density lipoprotein, *S-Cr* serum creatinine, *Ccr* clearance of creatinine, *U-Alb* urine albumin, *UACR* urine albumin to creatinine ratio.

*P < 0.05 when compared with CTL group, ^#^*P* < 0.05 when compared with HFD group.

### PA induced impaired autophagy followed by ER stress in HK2 cells

**Fig. 3:**
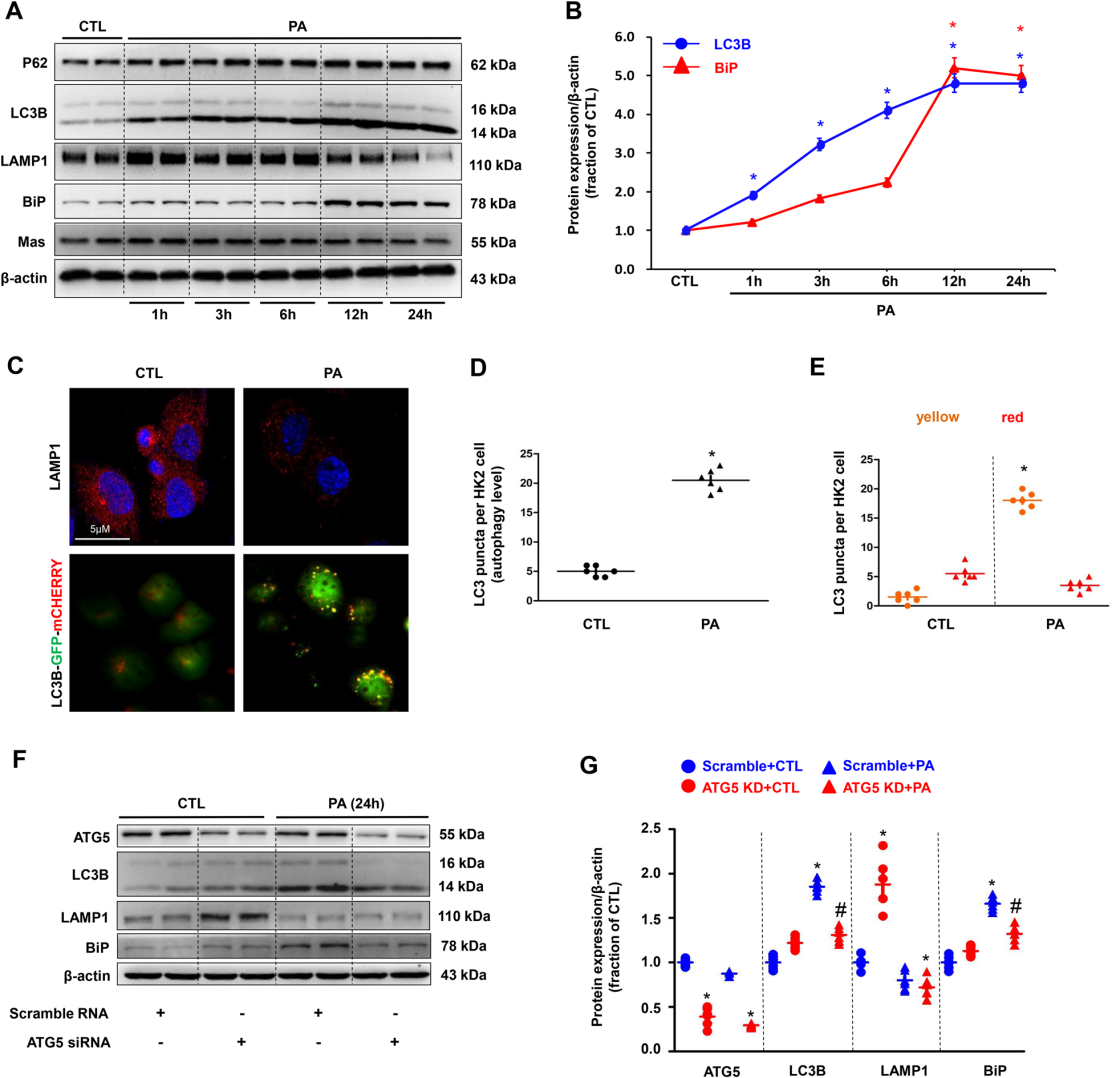
Palmitic acid (PA) induced impaired autophagy and ER stress in HK2 cells. **A** Representative immunoblots of autophagy protein markers (assessed by P62, LC3B and LAMP1) and activation of the ER stress (assessed by BiP) in cultured HK2 cells treated with PA (400 μM) for 1 h, 3 h, 6 h, 12 h, 24 h. β-actin was used as a loading control. **B** Dynamic changes of LC3B and BiP protein expression in HK2 cells treated with PA. **C** Confocal microscopy images of LAMP1 (red) and autophagic flux estimated by the formation of LC3B puncta in HK2 cells treated with or without PA. **D** The number of GFP-positive puncta was counted in HK2 cells. **E** Autophagy flux was detected by the ratio of yellow puncta (GFP and mCherry double-positive) in all LC3B puncta. High ratio of double-positive puncta in PA treatment group indicated PA induced impaired autophagy in HK2 cells. **F** and **G** Representative immunoblots and corresponding densitometry analysis of autophagy and ER stress protein markers in HK2 cells with ATG5 siRNA after PA treatment. Data are shown as mean ± SEM; *P < 0.05 compared with CTL; ^#^*P* < 0.05 compared with PA. The experiment was repeated three times.

To investigate the mechanism by which lipid induced tubular injury, the interaction of autophagy and ER stress evoked by PA was examined in cultured human proximal tubular HK2 cells. The protein abundance of LC3 and P62 was markedly upregulated even at the 1 h after PA (400 μM) treatment and progressively increased until 24 h, whereas LAMP1 expression was markedly and consistently decreased (Fig. 3A, B). After 24 h treatment with PA, immunofluorescence demonstrated decreased LAMP1 intensity (Fig. 3C) with enhanced LC3B dots, indications of autophagosomes (Fig. 3C) in HK2 cells. Quantification of total LC3 puncta showed a low basal level of autophagy in controls, PA significantly induced the formation of autophagosomes, as well as the maturation to autolysosomes in HK2 cells (Fig. 3D). The number of autophagosomes (yellow) per HK2 cell treated with PA was much more than those in controls (Fig. 3E), whereas the number of autophagolysosome (red) maintained (Fig. 3E). These data likely indicate an impaired autophagy and accumulation of autophagosome, induced by PA. Unlike early occurance of autophagy, the protein expression of BiP started to increase at the 6 h after PA treatment and dramatically increased at the 12 h and the 24 h (Fig. 3A, B), likely indicating a delayed ER stress followed autophagy.

To further examine the interaction between autophagy and ER stress, we inhibited ATG5, a key molecule in elongation and expansion of the phagophore in autophagy, in HK2 cells by using small interfering RNAs (siRNA) against ATG5. ATG5 depletion attenuated PA-induced upregulated LC3 and BiP protein expression in HK2 cells (Fig. 3F, G), indicating that impaired autophagy likely induced ER stress in HK2 cells after PA treatment.

3-Methyladenine (3-MA), an inhibitor of autophagosome formation, markedly inhibited protein abundance of LC3 and BiP (Supplementary data II Fig. S4A, B) in HK2 cells treated with PA, while choroquine (CQ), a late-stage autophagy inhibitor, effectively prevented autolysosome formation as seen in much increased LC3 and BiP protein expression (Supplementary data SII Fig. S4C, D). These data likely indicated that PA induced impaired autophagy (e.g., accumulation of autophagosome) and thus potentially stimulated ER stress. In this regard, rapamycin (RAPA), known to induce autophagosome formation, dramatically increased protein expression of LC3 and BiP in HK2 cells treated with PA (Supplementary data II Fig. S4E, F). Next, LC3B-GFP-mCHERRY adenovirus was used to detect the autophagy activity (Supplementary data II Fig. S4G). Quantification of total LC3 puncta showed that 3-MA dramatically reduced PA-induced LC3 puncta, while CQ and RAPA markedly increased the number of LC3 puncta (yellow and red) after PA treatment (Supplementary data II Fig. S4H, I). The autophagosome accumulation (yellow) was also increased by CQ or RAPA at 6 h after PA treatment, indicating that CQ or RAPA enhanced PA-induced autophagy stagnation (Supplementary data II Fig. S4J). In contrast, TUDCA, an ER stress inhibitor, failed to decrease protein abundance of LC3 in HK2 cells treated with PA, although BiP protein expression was significantly downregulated (Supplementary data II Fig. S5A, B), indicating that suppression of ER stress could not rescue impaired autophagy induced by PA. These findings strongly suggest that impaired autophagy and accumulation of autophagic bodies induced by PA may be the cause of ER stress and later cell death in HK2 cells.

### Mas-mediated PA-induced autophagy and ER stress in HK2 cells

**Fig. 4:**
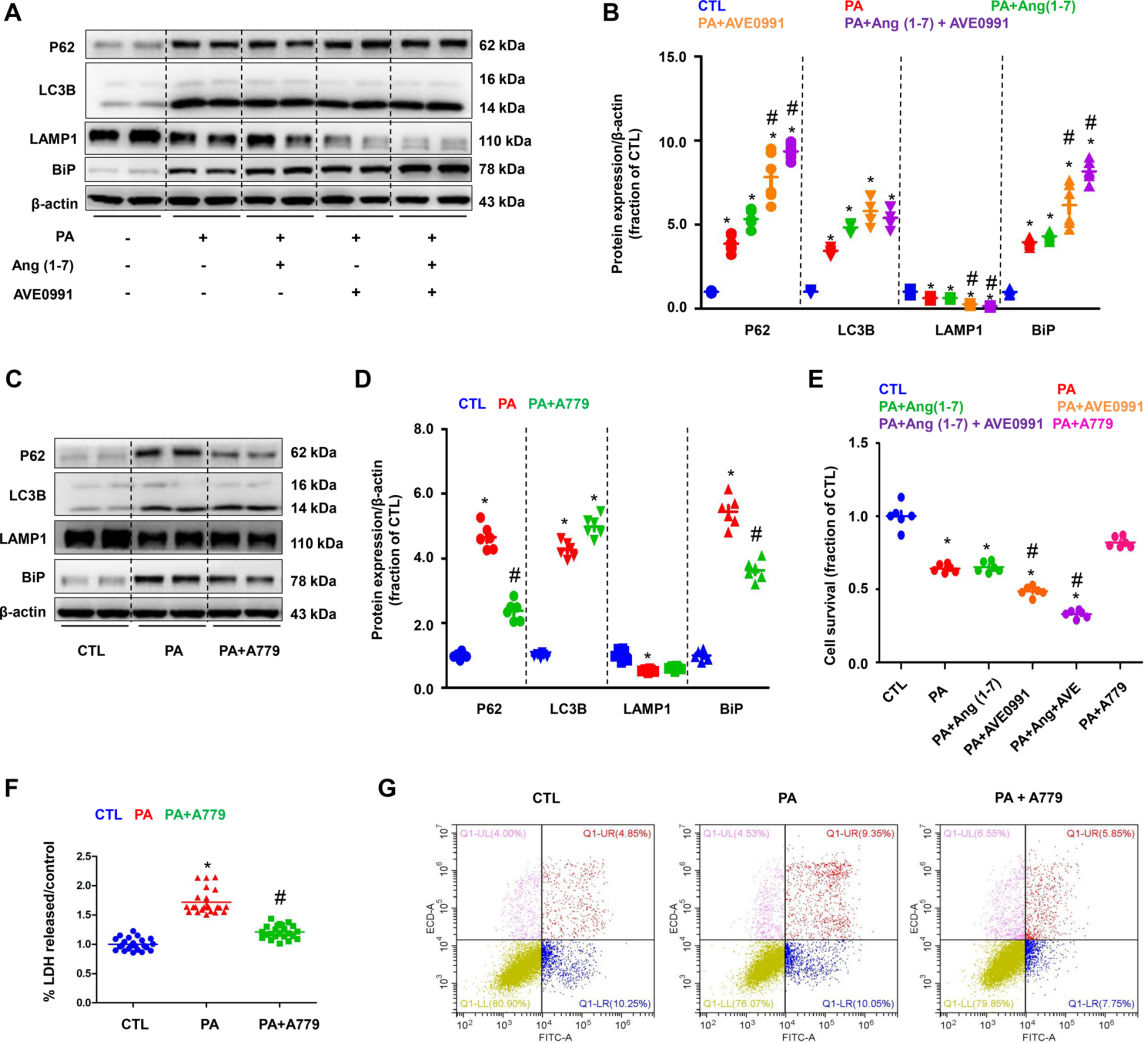
Mas-mediated PA-induced autophagy and ER stress in HK2 cells. **A** and **B** Representative immunoblots and corresponding densitometry analysis of protein markers of autophagy flux (assessed by P62, LC3B and LAMP1) and ER stress (assessed by BiP) in PA-treated HK2 cells pretreated with Mas agonists Ang (1–7) or AVE0991. **C** and **D** Representative immunoblots and corresponding densitometry analysis of protein markers of autophagy flux (assessed by P62, LC3B and LAMP1) and ER stress (assessed by BiP) in PA-overload HK2 cells pretreated with Mas antagonists A779. **E** Cell survival ratio of Mas activation or inhibition in HK2 cells treated with PA (400 μM) for 24 h assessed by CCK. **F** Lactate dehydrogenase (LDH) release by PA-overload HK2 cells pretreated with Mas antagonists A779. **G** Programmed cell death was detected by flow cytometry using Annexin V-FITC/PI apoptosis detection kit in PA-overload HK2 cells pretreated with Mas antagonists A779. Ang (1–7), angiotensin 1–7; CCK, cell counting kit 8. Data are shown as mean ± SEM; *P < 0.05 compared with CTL; ^#^*P* < 0.05 compared with PA. The experiment was repeated three times.

To determine the role of Mas receptor and its signaling pathway in PA-induced impaired autophagy and ER stress, Mas receptor agonists Ang (1–7), AVE0991, and an antagonist A779, were used. Ang (1–7) or AVE0991 dramatically enhanced PA-induced increased protein expression of P62, LC3B, and BiP, whereas LAMP1 protein expression was decreased (Fig. 4A, B). In contrast, A779 markedly decreased protein abundance of P62 and BiP induced by PA, while interestingly, A779 maintained the protein expression of LC3B and LAMP1 (Fig. 4C, D). These data suggests that activation of Mas receptor was able to worse the impaired autophagy induced by PA, leading to ER stress and cell death. Inhibition of Mas receptor improved autophagy and thus attenuated ER stress, at least partially, maintaining cellular homeostasis. Cell survival assay showed that activation of Mas receptor by AVE0991 or Ang (1–7) decreased cell survival, while inhibition of Mas receptor by A779 prevented cell damage induced by PA (Fig. 4E). Cellular injury was further assessed by measuring release of LDH to the medium. PA treatment caused a significant increase in LDH release in HK2 cells compared with controls. In the presence of a A779, LDH release was significantly lower (Fig. 4F). Annexin V-FITC/PI double staining showed that A779 at least partially prevented PA-induced cell apotosis in HK2 cells (Fig. 4G).

### Mas knockdown prevented PA-induced impaired autophagy and ER stress in HK2 cells

**Fig. 5:**
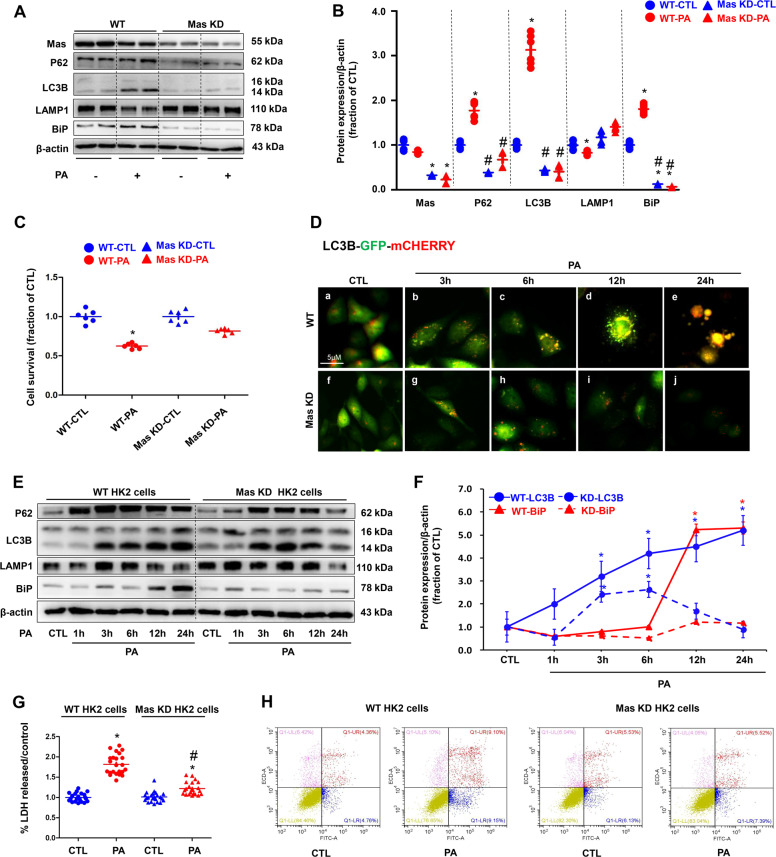
Mas knockdown (KD) prevented PA-induced impaired autophagy and ER stress in HK2 cells. **A** and **B** Representative immunoblots and corresponding densitometry analysis of protein markers of autophagy (assessed by P62, LC3B and LAMP1) and ER stress (assessed by BiP) in WT and Mas knockdown HK2 cells treated with PA (400 μM) for 24 h. **C** Cell survival ratio of WT HK2 cells and Mas knockdown HK2 cells were treated with either BSA or PA 400 μM for 24 h assessed by CCK. **D** Confocal microscopy images of WT and Mas knockdown HK2 cells treated with PA followed by transfection with LC3B-GFP-mCherry adenovirus for 3 h, 6 h, 12 h, 24 h. **E** Representative immunoblots of autophagy protein markers (assessed by P62, LC3B and LAMP1) and activation of the ER stress (assessed by BiP) in WT and Mas knockdown HK2 cells treated with PA (400 μM) for 1 h, 3 h, 6 h, 12 h, 24 h. β-actin was used as a loading control. **F** Dynamic changes of LC3B and BiP protein expression in WT and Mas knockdown HK2 cells treated with PA. **G** LDH release by WT HK2 cells or Mas knockdown HK2 cells that were treated with either BSA or PA 400 μM for 24 h. **H** Programmed cell death was detected by flow cytometry using Annexin V-FITC/PI apoptosis detection kit in WT HK2 cells and Mas knockdown HK2 cells that were treated with either BSA or PA 400 μM for 24 h. WT, wild type; Mas KD, Mas knockdown. CCK, cell counting kit 8. Data are shown as mean ± SEM; *P < 0.05 compared with CTL; ^#^*P* < 0.05 compared with PA. The experiment was repeated three times.

Gene expression of Mas in the renal tubular cells has been previously reported^6^. To further investigate the role of Mas receptor in PA-induced HK2 cell injury, Mas was knockdown in HK2 cells by using CRISPR/Cas9-guided genome editing. Blast results showed the binding sequence of gRNAs (Supplementary data II Fig. S6). Interestingly, compared to seen in wild type (WT) HK2 cells, Mas knockdown (KD) abolished PA-induced upregulation of P62/LC3B and BiP, while LAMP1 did not change (Fig. 5A, B). Cell survival assay showed that Mas KD was associated with better cell viability than that in WT HK2 cells under PA treatment (Fig. 5C), supporting the conception that Mas likely mediates PA-induced impaired autophagy, ER stress, and cell death. Autolysosome formation is a key step for autophagy, we next examined mRNA levels of markers of lysosome, including ACP2, UVRAG, and CTSB, in WT and Mas KD HK2 cells following PA treatment. In WT HK2 cells, PA significantly reduced mRNA levels of these three markers, whereas in Mas KD HK2 cells they maintained almost the same levels regardless of PA treatment (Supplementary data II Fig. S7). Immunofluorescence showed that more autophagosomes (green) were localized in lysosomes (red) in Mas KD HK2 cells than those in WT HK2 cells after 12 h treatment of PA (Fig. 5D). PA treatment induced an rapid and persistent impaired autophagy followed by a delayed ER stress in WT HK2 cells (Fig. 5E, F), but in Mas KD HK2 cells, PA increased the protein expression levels of LC3B and P62 only at the 3 h and the 6 h after treatment, which was downregulated at 12 h and nearly back to control level at 24 h after PA treatment (Fig. 5E, F). These were associated with unchanged, stable protein expression of BiP in Mas KD HK2 cells during 24 h treatment of PA (Fig. 5E, F). The release of LDH in PA-treated Mas KD HK2 cells was much lower than that in WT HK2 cells (Fig. 5G). Mas knockdown protected HK2 cells from PA induced cell apotosis (Fig. 5H). These data suggests that Mas receptor activation plays a key role in impaired autophagy and ER stress in PA-treated HK2 cells.

### Mas-mediated PA-induced elevated intracellular calcium levels in HK2 cells

Next, we investigated the possible molecular mechanism by which Mas mediates PA-induced autophagy and ER stress. In WT HK2 cells PA treatment was associated with markedly elevated intracellular calcium [Ca^2+^]_i_ as measured by FLUO-4 AM calcium probe, whereas in Mas KD HK2 cells cytosolic calcium levels were not as high as that in WT cells after PA treatment (Supplementary data II Fig. S8A). Moreover, AVE0991 increased and A779 inhibited the elevation of intracellular calcium levels induced by PA treatment in HK2 cells (Supplementary data II Fig. S8B, C). These observations suggest an involvement of intracellular calcium in Mas signaling pathway activated by PA.

### Mas-mediated mitochondrial injury induced by PA in HK2 cells

**Fig. 6:**
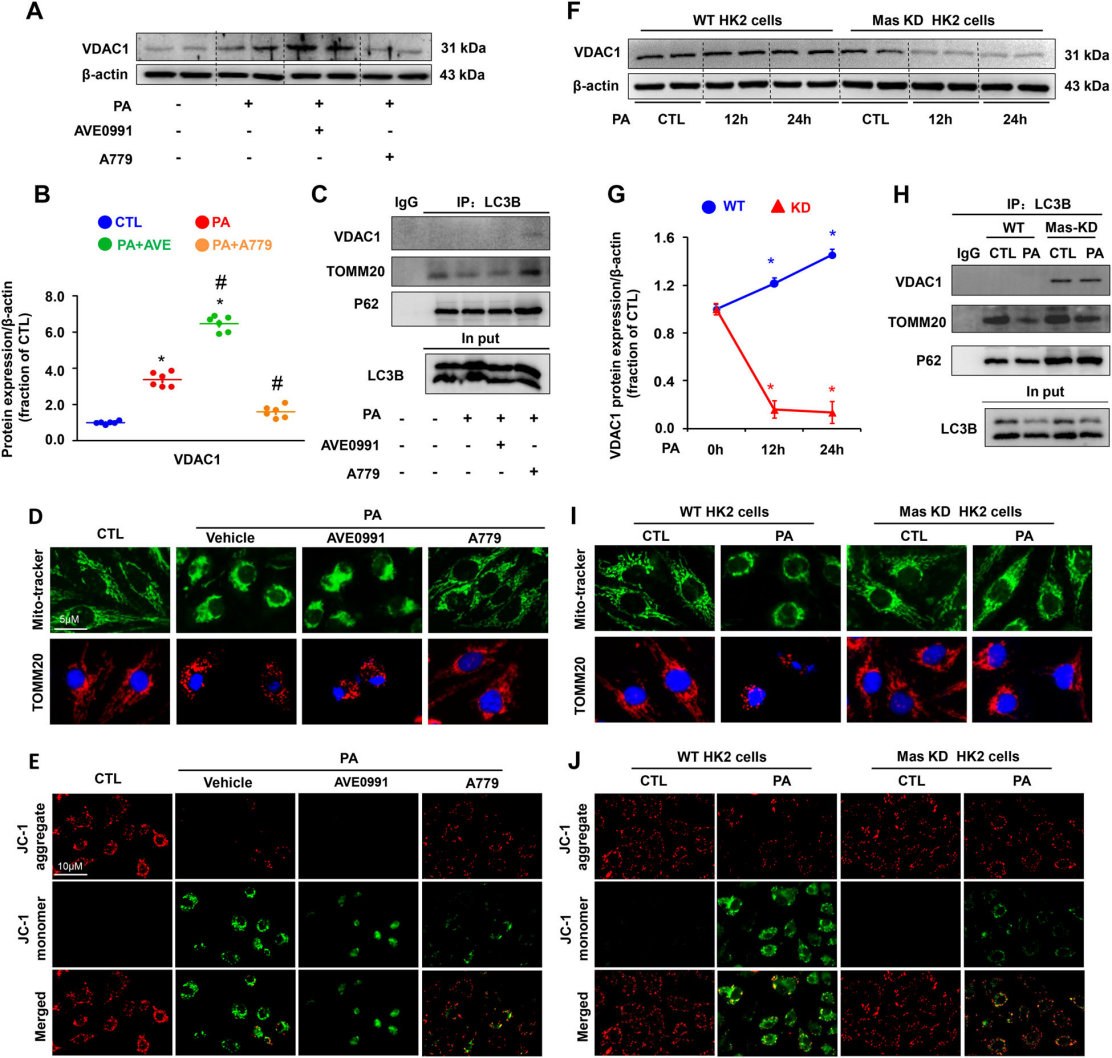
Mas activation was associated with increased VDAC1 expression and impaired mitochondrial morphology and potential in HK2 cells treated with PA. **A** and **B** Representative immunoblots and corresponding densitometry analysis of VDAC1 protein abundance in HK2 cells pretreated with AVE0991 or A779 followed by PA for 24 h. β-actin was used as a loading control. **C** VDAC1 and TOMM20 were detected by LC3 tag labeling in HK2 cells pretreated with AVE0991 or A779 followed by PA for 24 h. **D** Confocal microscopy images of Mito-tracker (green) and TOMM20 (red) in HK2 cells pretreated with AVE0991 or A779 followed by PA for 24 h. **E** Representative images of JC-1 staining showing JC-1 aggaregate (red) and monomer (green) in HK2 cells pretreated with AVE0991 or A779 followed by PA for 24 h. **F** and **G** Representative immunoblots and corresponding densitometry analysis of VDAC1 protein abundance in WT and Mas knockdown (KD) HK2 cells treated with PA for 12 h and 24 h. **H** Mitochondrial proteins VDAC1 and TOMM20 were detected by LC3 tag labeling in WT and Mas knockdown HK2 cells treated with PA for 24 h. **I** Confocal microscopy images of Mito-tracker (green) and TOMM20 (red) in WT and Mas knockdown HK2 cells treated with PA for 24 h. **J** Representative images of JC-1 staining showing JC-1 aggaregate (red) and monomer (green) in WT and Mas knockdown HK2 cells treated with PA for 24 h. WT, wild type; Mas KD, Mas knockdown Data are shown as mean ± SEM; *P < 0.05 compared with CTL; ^#^*P* < 0.05 compared with PA. The experiment was repeated three times.

Calcium overload in mitochondria affects β-oxidation of FFA, which is a major source of renal ATP synthesis, particularly in proximal tubular cells. PA treatment alone markedly increased protein abundance of VDAC1 (Fig. 6A, B), which is located on the outer membrane of mitochondria facilitating calcium transport into mitochondria. Interestingly, activation of Mas receptor by AVE0991 increased the protein expression of VDAC1, while A779 attenuated the expression of VDAC1 protein induced by PA (Fig. 6A, B).

We next labeled autophagy bodies in the cytoplasm by LC3B-Tag and examined protein changes of the mitochondrial marker proteins and autophagy-related proteins. The protein expression of TOMM20, a mitochondrial membrane marker in autophagy bodies, was dramatically decreased after PA treatment, which was aggravated by Mas activation (Fig. 6C) and alleviated by Mas inhibition (Fig. 6C). The interaction between VDAC1 and LC3B was found increased with Mas inhibition (Fig. 6C), which likely indicates more removel of damaged mitochondria by autophagy. By staining with a mitochondrial probe Mito Tracker and TOMM20 in HK2 cells, it was demonstrated that more damaged mitochondria accumulated around nuclei with PA treatment, which was markedly prevented by Mas inhibition, whereas the Mas activation by AVE0991 appeared to aggravate mitochondrial damages and abnormal distributions in HK2 cells (Fig. 6D). Mitochondrial membrane potential was examined by using JC-1 in HK2 cells treated with PA. JC-1 exists as either a green-fluorescent monomer at depolarizing mitochondrial or a red-fluorescent aggregate at polarizing mitochondria. In control cells with normal mitochondrial membrane potential, mitochondrial were marked by punctate red fluorescence of JC-1 with no green signal detected, on the contrary, persistent PA treatment led to dramatical decreases of red JC-1 signals, which displayed intensely diffused green monomer fluorescence signals (Fig. 6E). The switch from red to green JC-1 signal indicated the loss of mitochondrial membrane potential in these PA-treated HK2 cells. Of interest, inhibition of Mas by A779 significantly suppressed mitochondrial depolarization during prolonged PA treatment, while activation of Mas by AVE0991 showed mild aggravation on mitochondrial depolarization (Fig. 6E).

As expected, Mas knockdown attenuated PA-induced increased expression of VDAC1 protein when compared to WT cells (Fig. 6F, G). Mas knockdown was also associated with more interactions between VDAC1 and LC3B than WT cells (Fig. 6H). Mas knockdown improved mitochondrial distributions (Fig. 6I) and mitochondrial depolarization (Fig. 6J) when compared with WT cells after PA treatment. These findings suggest an involvement of Mas signaling in mitochondrial dysfunction induced by PA in HK2 cells.

### Mas-mediated abnormal ubiquitination degradation of VDAC induced by PA in HK2 cells

**Fig. 7:**
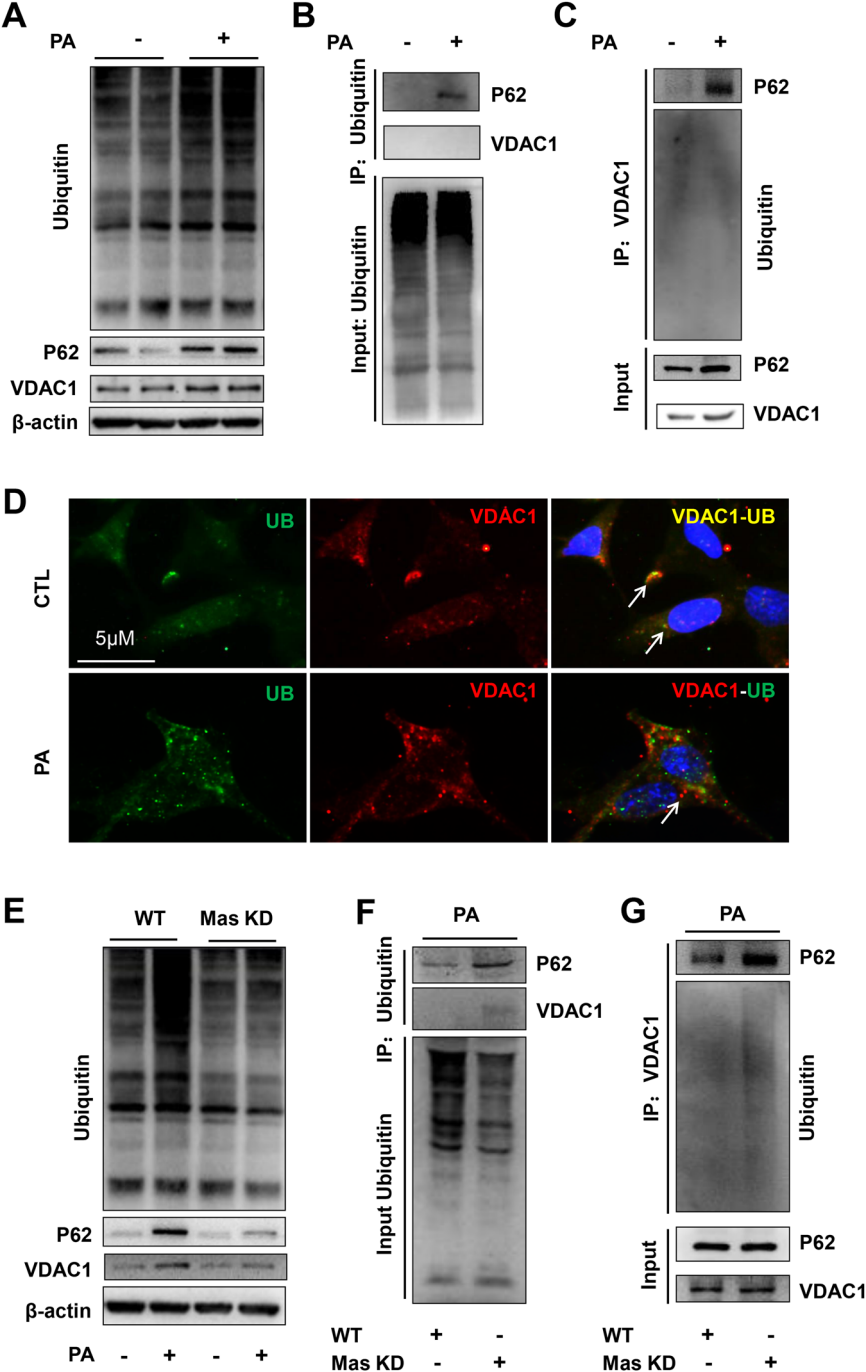
Mas knockdown (KD) improved incomplete ubiquitination of VDAC1-P62-ubiquitin complex in HK2 cells treated with PA. **A** Protein expression of VDAC1, P62 and ubiquitin in HK2 cells treated with PA for 24 h. **B** and **C** Immunoprecipitation assay showing the interaction among VDAC1, P62, and ubiquitin in HK2 cells with or without PA treatment. **D** Confocal microscopy images of VDAC1 (red) and ubiquitin (UB) (green) in HK2 cells treated with or without PA, showing more colocalization of VDAC1 and ubiquitin in controls than that in PA-treated cells. **E** Protein expression of VDAC1, P62 and ubiquitin in WT and Mas KD HK2 cells with or without PA for 24 h. **F** and **G** Immunoprecipitation assay showing the interaction among VDAC1, P62 and ubiquitin in WT and Mas KD HK2 cells with or without PA treatment. WT, wild type; Mas KD, Mas knockdown. The experiment was repeated three times.

STRING database and other evidence indicate interactions among VDAC1, LC3/P62 and ubiquitin, in particular, the interaction among VDAC1, ubiquitin, and P62 was recently shown to mediate mitophagy pathway^27,33^. We next investigated whether PA treatment affects interactions among VDAC1, P62 and ubiquitin. The expression of VDAC1, P62 and ubiquitin proteins was clearly increased with PA treatment (Fig. 7A). Co-immunoprecipitation assay demonstrated that PA treatment induced a linkage of P62 and ubiquitin (Fig. 7B) and an interaction between VDAC1 and P62 (Fig. 7C). Colocalization of ubiquitin and VDAC1 was obviously observed in control HK2 cells, but not in PA-treated HK2 cells (Fig. 7D), likely suggesting an incomplete ubiquitination of VDAC1 induced by PA. Mas knockdown suppressed PA-induced upregulation of ubiquitin, VDAC1 and P62 protein (Fig. 7E), but promoted the interaction between P62 and VDAC1 (Fig. 7F), and the interaction between VDAC1 and ubiquitin (Fig. 7G), respectively. These results suggested that Mas signaling probably mediates an incomplete ubiquitination of VDAC1-P62 complex in HK2 cells treated with PA.

**Fig. 8:**
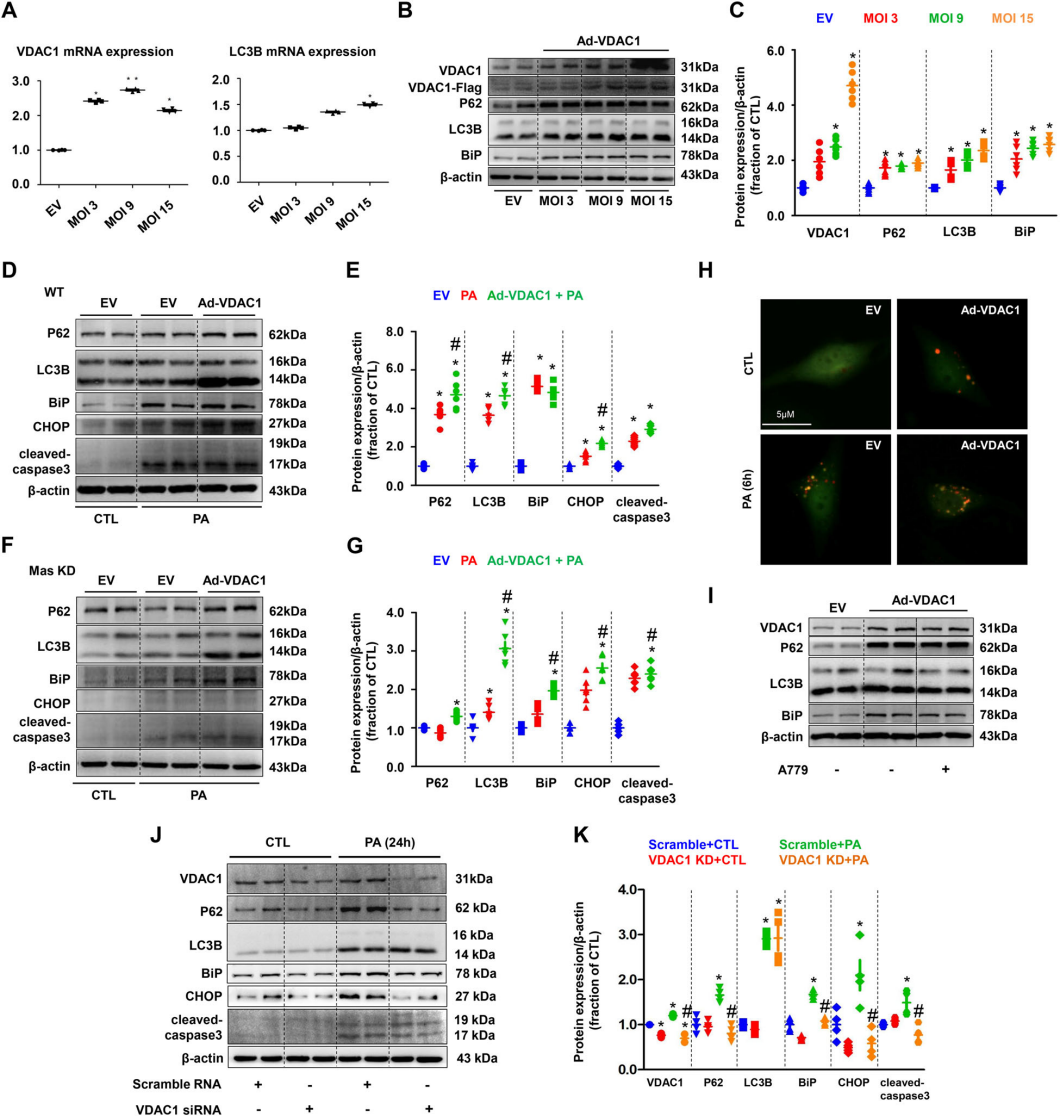
VDAC1 was involved in PA-induced impaired autophagy, ER stress and cell death in HK2 cells. **A** mRNA levels of VDAC1 and LC3B were examined by quantitative RT-PCR in empty vector (EV) and adenovirus encoding VDAC1 (Ad-VDAC1) transfected HK2 cells. **B** and **C** Representative immunoblots and corresponding densitometry analysis of VDAC1, P62, LC3B, and BiP protein abundance in HK2 cells transfected with Ad-VDAC1. β-actin was used as a loading control. **D** and **G** Representative immunoblots and corresponding densitometry analysis of protein markers of autophagy, ER stress and apoptosis in PA-treated WT and Mas knockdown (KD) HK2 cells transfected with Ad-VDAC1. β-actin was used as a loading control. **H** Confocal microscopy images of LC3B puncta formation in Ad-VDAC1 transfected HK2 cells treated with PA (400 μM) for 6 h. **I** Representative immunoblots and corresponding densitometry analysis of VDAC1, P62, LC3B, and BiP protein abundance in EV or Ad-VDAC1 transfected HK2 cells treated with A779. **J** and **K** Representative immunoblots and corresponding densitometry analysis of VDAC1, P62, LC3B, BiP, CHOP and cleaved-caspase-3 protein abundance in PA-treated HK2 cells transfected with VDAC1 siRNA. β-actin was used as a loading control. EV, empty vector; Ad, adenovirus. Data are shown as mean ± SEM; *P < 0.05 compared with EV + CTL; ^#^*P* < 0.05 compared with EV+PA. The experiment was repeated three times.

In order to further determine the role of VDAC1 in PA-induced autophagy and ER stress, Ad-VDAC1 overexpression virus was transfected in PA-treated HK2 cells. Overexpression of VDAC1 was associated with increased mRNA gene levels of LC3B (Fig. 8A) and increased the protein expression of P62, LC3B, and BiP (Fig. 8B, C). Overexpression of VDAC1 caused upregulation of autophagy, ER stress and apoptosis markers, regardless of PA treatment (Fig. 8D, E). In Mas KD HK2 cells, PA treatment generally maintained or slightly increased protein abundance of these markers, overexpression of VDAC1 evoke dramatically increased LC3B, BiP, CHOP, and cleaved-caspase-3 (Fig. 8F, G). Immunofluorescence with LC3B-GFP-mCHERRY adenovirus showed impaired autophagy in HK2 cells overexpressed VDAC1 with or without PA treatment (Fig. 8H). A779 failed to decrease expression of above proteins in HK2 cells with overexpressed VDAC1 (Fig. 8I). In contrast, VDAC1 knockdown by siRNA prevented impaired autophagy and ER stress induced by PA in HK2 cells (Fig. 8J, K). Taken together, our data suggest that incomplete ubiquitination of VDAC1 and thus persistent expression of VDAC1 in mitochondria may result in impaired autophagy, ER stress, and apoptosis in HK2 cells treated with PA, independ on Mas pathway.

## Discussion

In the present study, Mas receptor signaling pathway was found involved in high-fat diet or PA-induced kidney injury, likely through activating autophagy and ER stress. Elevated intracellular calcium and incomplete ubiquitination-related degradation of VDAC1-mediated PA-induced impaired autophagy, ER stress and apoptosis in tubular epithelial cells. These data are novel and helpful to elucidate the molecular mechanism of lipid-induced kidney injury.

The components of the ACE2/Ang-(1–7)/Mas axis have important roles in metabolic regulation. Genetic deletion of Mas in FVB/N mice leads to a metabolic syndrome, like hypertension, increase in blood glucose and triglycerides and cholesterol^16^. However, ablation of Mas in C57BL/6 mice produced only minor changes in glucose and lipid metabolism, although cholesterol levels were elevated in old mice^16^. Diet-induced obesity was less pronounced in Mas-knockout FVB/N than in widetype mice^17^. Similar to this, our Mas^−/−^ mice in C57BL/6 showed unchanged lipid profile. Interestingly, HFD only caused mild metabolic changes in these mice when compared to wild type, indicating a resistance of Mas deletion to metabolic syndrome in this mouse strain. The protective role of Ang (1–7)/Mas in the heart and vessels are generally accepted, while the known actions of Ang (1–7)/Mas in the kidney are shown more complex and unpredictable (see review by Santos ref. ^11^). Our recent study revealed that PA or HFD-induced tubular injury likely through activating local RAS, leading to angiotensin-II-associated ER stress and apoptosis^3,4^. We originally assumed that Ang (1–7)/Mas may excert a protective effect in PA or HFD-induced tubular injury, unexpectedly, Mas deletion or Mas blockade in mice with HFD actually prevented tubular injury, likely through preventing autophagy, ER stress, and apoptosis in the kidney. This is in contrast to the well known counteraction of Ang (1–7)/Mas to angiotensin-II axis in the cardiovascular system. Although the mechanism is still unknown, it is essential to be aware of any unfavorable or deleterious effects of Mas activation in the kidney, when the therapeutic potential of ACE2/Ang (1–7)/Mas in metabolic or cardiovascular diseases is widely noticed.

PA or HFD is known to activate autophagy^23^ and to induce ER stress^3^ in proximal tubular epithelial cells. A large number of evidences has shown that ER stress may induce autophagy, namely ER stress-mediated autophagy^19,21^. However, in the current study, we demonstrated a possibility that PA/HFD-induced impaired autophagy may cause ER stress and apoptosis in tubular epithelial cells. PA treatment induced elevated, persistent autophagy much earlier than ER stress in HK2 cells and cultured PTCs. Interestingly, when ATG5 was inhibited, PA failed to induce both autophagy and ER stress. Furthermore, inhibition of autophagosome formation by 3-MA attenuated PA-induced ER stress, whereas inhibition of autolysosome formation by CQ or induction of autophagy by RAPA was associated with increased ER stress in HK2 cells treated with PA. These data indicates a casual association between autophagy and ER stress, although the underlying mechanism remains to be elucidated. As a pro-survival mechanism, autophagy may protect cells from injury and death caused by PA, but persistent, unresolved autophagy may be deleterious. In autophagy, autophagosomes deliver cytosolic content to lysosomes forming autolysosome, where autophagosomal-sequestered cargo is degraded by lysosomes. A recent study elegantly demonstrated that lipid overload basically stimulates autophagy and long-term lipid overload places a burden on the lysosomal system, resulting in stagnant autophagic flux^23,24^. In the current study, more autophagosomes, but not autophagolysosomes, were observed in our PA-induced tubular cells, indicating an autophagy stagnation. Given that autophagosome accumulation in PTCs can be induced by lipid overload, cellular functions may be jeopardized in pathologic settings in which properly reduced autophagosome should exert a protective role. Therefore, 3-MA, but not CQ, inhibited autophagy and, at the same time, rescued ER stress after PA treatment. In contrast, when PA-induced ER stress was suppressed by TUDCA, autophagy was still maintained. Taken together, these data strongly suggest that autophagylysosome pathway is disrupted by lipid, resulting in the accumulation of abnormal proteins. Unstable proteostasis and autophagy in the tubular compartment may increase burden of ER, causing ER stress and apoptosis.

How does PA induce autophagy and ER stress? Our previous studies showed that angiotensin-II plays a role in mediating in PA-induced ER stress and apoptosis in tubular cells^4^, suggesting a role of local RAS. Ang (1–7) and its G-protein coupled receptor Mas has been shown to counteract the classical RAS, conferring beneficial effects^5,6^. Surprisingly, our data demonstrated an involvement of Mas receptor pathway in PA/high-fat induced renal tubular cell injury. Mas activation by agonist Ang (1–7) or AVE0991 actually failed to attenuate PA-induced autophagy and indeed aggravated ER stress in HK2 cells, whereas Mas antagonist A779 clearly decreased autophagy and ER stress. Importantly, Mas knockdown or Mas deletion in mice fed HFD markedly attenuated PA-induced impaired autophagy and ER stress in HK2 cells or cultured PTCs when compared to wild type. Mas knockdown was also associated with less autophagosomes than wild type HK2 cells after PA treatment, likely indicating more lysosomes with normal function effectively fusing to autophagosomes and degrading cargos. Time-course data also showed very slightly increased LC3B and BiP protein expression in Mas knockdown cells or Mas^−/−^ PTCs after PA treatment in contrast to dramatical upregulation of two protein expression in wild type cells. These data suggested that inhibition of Mas pathway improve PA-induced impaired autophagy and thus attenuate ER stress, potentially, preventing ER-associated cell death.

The next question is which potential mechanism mediates Mas signaling pathway after PA treatment. Mas is G-protein coupled receptor, which acts through cAMP/PKA pathways to regulate and phosphorylate its substrates on specific serine and threonine residues, e.g., phosphorylation of calcium channel. Recent studies demonstrated that Ang (1–7) increased intracellular calcium levels in microperfused proximal tubular cells, which was markedly inhibited by A779^9,10^. The stimulated increase of [Ca^2+^]_i_ by Ang (1–7) was likely attributed to influx of extracellular calcium, since a membrane impermeable calcium chelator that binds extracellular calcium ions markedly abolished Ang (1–7)-induced increased [Ca^2+^]_i_^9^. PA treatment was associated with increased [Ca^2+^]_i_ in HK2 cells, which was accelerated by Mas activation with AVE0991 and attenuated by Mas inhibition with A779. In HK2 Mas knockdown cells, PA evoked less increased [Ca^2+^]_i_ compared with wild type cells. These data likely suggested Mas-mediated increase of intracellular calcium may be involved in PA-induced cell injury,.

Imbalance in the control of [Ca^2+^]_i_ can lead to mitochondrial Ca^2+^ overload and ultimately, to toxic effects, e.g., apoptosis. VDAC1 in the outer mitochondrial membrane mediates the transport of Ca^2+^ into and out of mitochondria, playing an important role in apoptosis^34^. VDAC1 overexpression increases mitochondrial Ca^2+^ concentration, while silencing of VDAC1 expression attenuates mitochondrial Ca^2+^ uptake and cell apoptosis^35^. Very lately overexpression and mistarget of VDAC1 to the plasma membrane was found to cause ATP release and cell apoptosis^36^. In our study, PA treatment caused upregulated VDAC1 protein expression, which, together with increased [Ca^2+^]_i_, may enhanced Ca^2+^ uptake by mitochondria, resulting in altered mitochondrial morphology, mitochondrial potential. Interestingly, upregulation of VDAC1 and altered mitochondrial markers was markedly prevented by Mas inhibition or knockdown in HK2 cells. In mice with high-fat diet, increased VDAC1, autophagy and ER stress makers was also prevented by A779, or by Mas deletion, indicating Mas activation mediates PA/high-fat-induced mitochondrial dysfunction, autophagy and ER stress.

Importantly, besides increased protein expression of VDAC1, we found a failure of VDAC1 ubiquitination degradation by binding with ubiquitin and P62, which was supposed to further aggravate mitochondrial dysfunction. The expression of VDAC1, P62 and ubiquitin proteins was clearly increased with PA treatment, however, interactions between ubiquitin and VDAC1, P62 and VDAC1 after PA treatment was much weak, likely indicating that an adaptor of damaged mitochondria is hardly identified by the P62 and thus leading to an incomplete ubiquitination of VDAC1. Again, more interactions among ubiquitin, P62 and VDAC1 were observed when Mas was knockdown in HK2 cells, suggesting that Mas signaling mediate an incomplete ubiquitination of VDAC1-P62-ubiquitin complex, which may be critical in leading to impaired autophagy and ER stress in HK2 cells treated with PA. Furthermore, inhibition of VDAC1 prevented impaired autophagy and ER stress induced by PA in HK2, whereas overexpression of VDAC1 in HK2 cells was also associated with impaired autophagy, increased ER stress and apoptosis, which was not able to be rescued by Mas knockdown or inhibition, indicating VDAC1 ubiquitination is one of downstream events in Mas signaling pathway.

In one hand, increased calcium uptake, through PA-assoicated incomplete ubiquitinated and persistent expression of VDAC1, leads to injured mitochondria that are assumed to be eliminated by autophagy, as clearance of damaged mitochondria prevents kidney injury^37^. However, PA caused an impairment of lysosomal acidification resulting in accumulation of autophagosomes^23^ containing damaged organelles (e.g., mitochondria) and cellular debris. On the other hand, PA-induced elevated intracellular calcium itself promotes autophagy via multiple mechanisms. Increasing cytosolic Ca^2+^ with exogenously introduced Ca^2+^ can induce autophagy at early time points without altering the ER condition^38,39^. Another study demonstrated that increased cytosolic calcium triggers autophagy as evidenced by more formation of autophagosomes^40^. The increase in the number of autophagosomes induced by elevated cytosolic calcium was due to the increased formation of autophagosomes rather than their defective turnover^40^. Accumulation of autophagosomes due to either increased formation or decreased fusion with lysosome may alter cellular proteostasis and increase burden of ER to handle protein production, leading to ER stress. In addition, both ROS released from mitochondria^41^ and elevated cytosolic free Ca^2+ ^^42^, after PA treatment may also be involved in increased ER stress. Eventually, when ER stress is unsolved and ER is so severely impaired, programmed cell execution is initiated, e.g., apoptosis, to sustain body homeostasis.

**Fig. 9:**
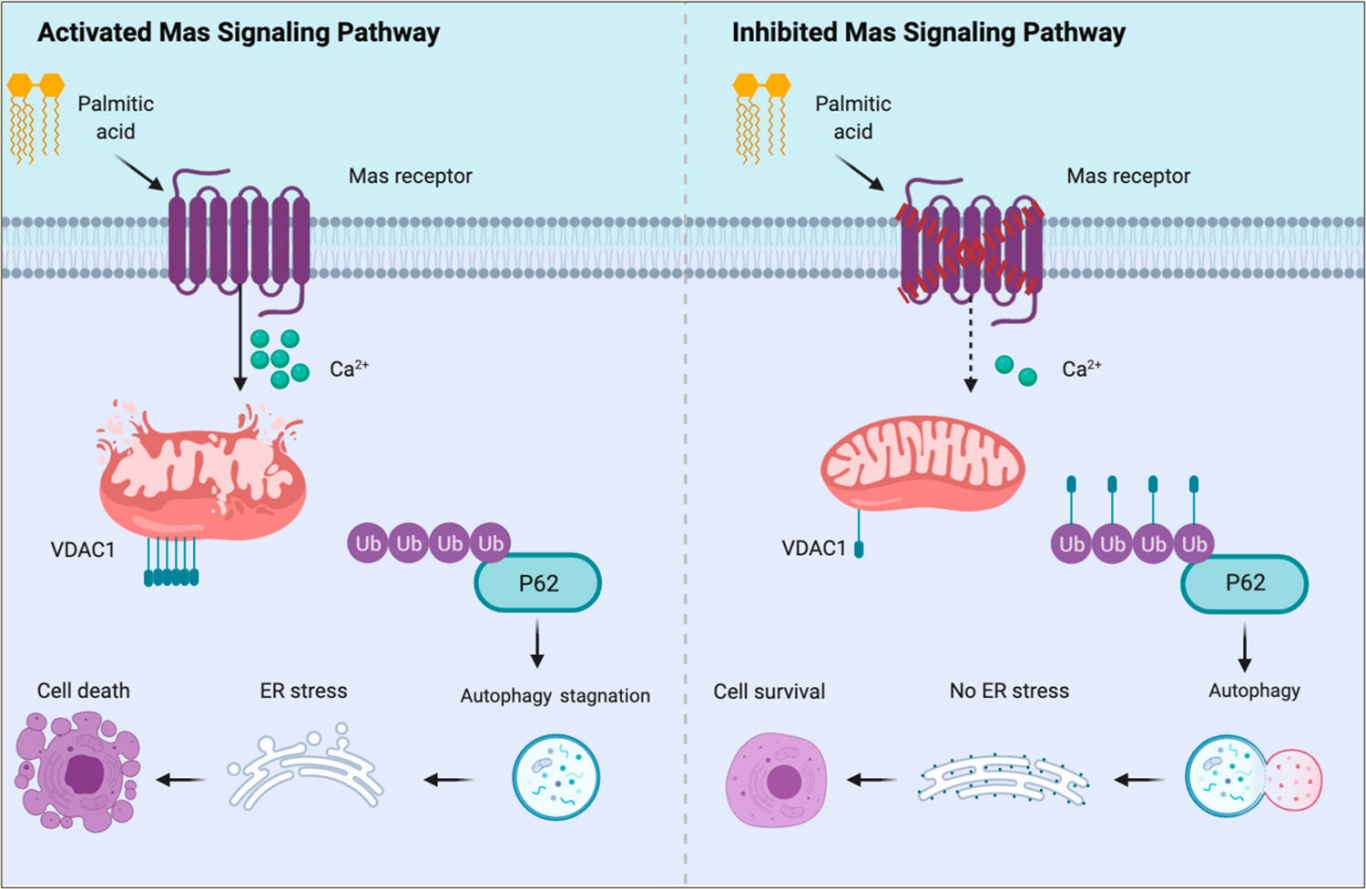
A potential mechanism by which Mas mediates PA/high-fat-induced impaired autophagy flux and ER stress in proximal tubular cells. Activation of Mas resulted in increased intracellular calcium levels, via incompletely degradated VDAC1 due to failure in binding to P62 and ubiquitin, causing mitochondrial damage and accumulation of autophagosomes, which induces ER stress and apoptosis.

Taken together, the current study demonstrated that PA/high-fat diet caused renal tubular injuries through Mas signaling pathway. Activation of Mas pathway resulted in increased intracellular calcium levels, via incompletely degradated VDAC1, causing mitochondrial damage and impaired autophagy, which may result in ER stress and apoptosis (Fig. 9). The study has implication in understanding mechanism of lipid-induced kidney injury and indicates that Mas blockade might be a reasonable way to ameliorate obesity-associated kidney diseases. There are still some questions need to be answered, for example, how does PA activate Mas on the plasma membrane of tubular epithelial cells? Could the conformation changes of Mas receptor on plasma membrane or changes of membrane components after PA/High-fat diet mediate downstream signaling of Mas resulting in tubular injuries? Apparently, as the role of Mas in renal physiology and pathophysiology is complicated, more studies, particularly those on specific Mas knockout in proximal tubules, are definitely warranted.

## Methods and materials

### Mice

The animal study was approved by the Animal Care and Use Committee of Sun Yat-sen University (Ethics Committee of ZSSOM on Laboratory Animal Care No. 2019-0160XS; Guangzhou, China). Mice with Mas gene deletion on a background of C57BL/6 were obtained from VIEWSOLID BIOTECH, Beijing, China. For identification of the genotypes, DNA extracted from tails of both Mas^+/+^ and Mas^−/−^ mice were amplified by PCR and then used for agarose gel electrophoresis or DNA sequence analysis. Ten-week-old male C57BL/6 (WT), Mas deletion mice were enrolled in these experiments. All mice were housed in an animal facility with a 12 h light–dark cycle and water ad libitum. Animals were randomly numbered and assigned into different groups and no mice were excluded at the end of experiments (*n* = 6 in each group). Researchers were not blinded during experiments and data analysis. The mice were either fed with a low-fat diet (10% of total calories from fat) or a high-fat diet (HFD) (60% of total calories from fat) (Guangdong Medical Lab Animal Center, China) for 12 week. For the A779 experiment, 10-week-old male C57BL/6 mice were performed unilateral nephrectomy 1 week before starting HFD for 12 weeks. Some of mice fed HFD have been intraperitoneally injected A779 (Shelleck, USA) (1 mg/kg body weight/day) dissolved in saline once a day. Mice were placed in metabolic cages for 24 h urine collection at the 12th week. All mice were anesthetized with pentobarbital, and the kidneys were rapidly excised from mice to perform biochemical and histological examinations, as described in following sections. Blood and urine biochemistry was measured at the 1st affiliated hospital, Sun Yat-sen University.

### Cell culture and treatment

HK2 cells, an immortalized human kidney proximal tubular epithelial cell line, was purchased from ATCC. HK2 cells were grown in DMEM containing 10% FBS and 1% penicillin & streptomycin and maintained at 37 °C in 5% CO_2_ atmosphere. Mas-deficient and wild type mouse PTCs were prepared as previously described^31^. In brief, kidney PTCs were isolated from 10-week-old Mas^+/+^ and Mas^−/−^ mice. Cells were cultured in DMEM containing 10%FBS at 37 °C in 5% CO_2_ atmosphere. The experiment was repeated three times.

### Palmitic acid was prepared as previously described^3^

Other inhibitor or agonist treatment: To test the effects of autophagy inhibitor, HK2 cells were pretreated with 10^−5 ^M chloroquine (Sigma, USA) or 10^−3 ^M 3-methyladenine (MCE, China) or 10^−5^ M rapamycin (MERYER, China) for 30 min, and then treated with PA for 24 h. To determine the role of Mas receptor, HK2 cells were pretreated with 10^−5 ^M Ang (1–7) (MCE, China), 10^−5^ M AVE0991 (MCE, China) or 10^−5^ M A779 (MCE, China) for 30 min, and then treated with PA for 24 h.

### Plasmid and adenovirus, siRNA transfection

#### Plasmid transfection

Mas knockdown plasmid was constructed as described previously^43^. Briefly, knockdown of Mas in HK2 cells was performed by CRISPR/Cas9-guided genome editing. Two of 20 nucleotide sgRNA sequences: sgRNA1 5′-TATGAGTATTGGTCGACCTT-3′ (160328740-160328760); sgRNA2 5′-CAATTGTCTTTCTGGCGCCG-3′ (160328911-160328931) were designed using sgRNA CRISPR design tool online (http://crispr.mit.edu) and cloned into the pSpCas9(BB)-2A-puro (PX459) plasmid (Addgene, catalog no.62988). After cloning, plasmids were purified and verified by sequencing. HK2 cells were seeded into six-well plates at 70% confluence and transfected with PX459 plasmid encoding a target-specific sgRNA using Lipofectamine 3000 (Invitrogen) according to the manufacturer’s instructions. Puromycin was then added to select the transfected cells after 24 h transfection. KD efficiency for Mas was assessed by western blot.

#### Adenovirus transfection

Cells were transfected with Adenovirus encoding VDAC1 (Ad-VDAC1) (ASBIOTECH, Guangzhou, China) or control virus (Ad-Flag) as described previously^43^. Cells were transfected with Adenovirus encoding VDAC1 (Ad-VDAC1) (ASBIOTECH, Guangzhou, China) or control virus (Ad-Flag) as described previously. Cells were seeded in 35 mm dishes at 4*10^5^ cells/dish and grown in DMEM supplemented with 10% FBS. When cells reached 80% confluence, Ad-VDAC1 or Ad-Flag were used to infect cells at an optimal multiplicity of infection (MOI = 9) and the transfection efficiency reaches 70%. Transfected cells were harvested at indicated time points for further analysis.

#### siRNA transfection

Cells were transfected at 60% confluence in DMEM without FBS using Lipofectamine 3000 (Invitrogen) according to the manufacturer’s instructions. The final ATG5 or VDAC1 siRNA (RIBOBIO, Guangzhou, China) concentration was 100pM. ATG5 or VDAC1 suppression was confirmed by western blot.

### Cell viability assays

Culture cells were seeded into 96-well plates at a density of 5000 cells per well. To analysis the role of MAS receptor in PA induced cells injury, the cells were treated with 10 μM A779, 10 μM Ang (1–7) and 10 μM AVE0991 or not for 24 h during PA treatment. For the detection of the role of MAS KD in PA induced cells injury, WT and MAS KD HK2 cells were treated with PA for 24 h. After treatments, the cell viability was determined by using the Cell Counting Kit-8 cell viability kit according to the manufacturer’s instructions (Beyotime, Shanghai, China). The absorbance at 450 nm was measured using a microplate reader (BIOTECK, USA). Relative survival rate in the presence of Ang (1–7), AVE0991, or A779 were normalized to the untreated controls after background subtraction..

### LDH release assay

The cells were plated into a 96-well plate. After treatments, the supernatant of each well was collected; the LDH release in the supernatant was determined by using a LDH reagent kit (Beyotime, Shanghai, China).

### Annexin V-FITC/PI apoptosis flow cytometry

The extent of programmed cell death was detected by flow cytometry using Annexin V-FITC/PI apotosis detection kit (BD Pharmingen, USA). Briefly, HK2 cells were harvested and washed twice with PBS after incubation with or without PA for 24 h. The cells were centrifuged at 1500 rpm/min for 5 min and stained with Annexin V-FITC and PI in the dark, followed by cytometry.

### Measurement of autophagy, intracellular calcium levels, and mitochondrial membrane potential

Cells cultured on 35 mm glass were subjected to the confocal analysis. Autophagy ratio was determined by transfected LC3B-GFP-mCHERRY virus (Beyotime, Shanghai, China) for 24 h at 37 °C. Autophagy dynamics was analyzed in HK2 cells expressing GFP-mCHERRY-LC3. The numbers of yellow (colocalizing GFP-LC3 with mCHERRY-LC3) puncta per cell and total red (mCHERRY-LC3) puncta per cell were counted separately using ImageJ. The number of autophagosomes was indicated by yellow puncta and the number of autolysosomes was obtained by subtracting yellow puncta from total red puncta. The number of autolysosomes was further divided by the total number of mCHERRY-LC3 puncta to indicate the autophagic flux rate. The mitochondrial morphology was determined by staining with 50 nM of Mito-Tracker (Beyotime, Shanghai, China) for 20 min at 37 °C, and mitochondrial membrane potential was examined in cells incubated with 5,5′,6,6′-tetrachloro-1,1′,3,3′- tetraethylbenzimidazolylcarbocyanine iodide (JC-1) (Med Chem Express, Shanghai, China) in medium for 1 h. After treatment, live cells were directly examined by fluorescence microscopy. To determine the intracellular calcium level, cells were incubated with 10 nM FLUO-4 AM probe for 30 min at 37 °C before subjected to cytoflow analysis. The experiment was repeated three times.

### Western blotting and co-immunoprecipitation (IP) studies

HK2 cells or kidney cortex were lysed in protein lysis buffer for 15 min on ice before protein was extracted. Immunoblotting was performed by electrophoresis and incubation with primary antibodies against P62 (1:1000, 5114, Cell signaling technology, USA), LC3B (1:1000, 2775S, Cell signaling technology, USA), BiP/GRP78 (1:1000, 3177S, Cell signaling technology, USA), CHOP (1:1000, 2895S, Cell signaling technology, USA), cleaved caspase-3 (1:1000, 9661S, Cell signaling technology, USA), VDAC1 (1:500, BM4279, Proteintech, USA), TOMM20 (1:500, BM4366, Proteintech, USA), Mas (1:1000, ab156018, Abcam, USA), LAMP1 (1:1000, ab24170, Abcam, USA), followed by the addition of horseradish peroxidase-labeled secondary antibodies. The blots were visualized ECL detection systems. Densitometric analysis was performed using AlphaEase Software. The experiment was repeated three times.

The samples subjected to IP assay were incubated with an anti-VDAC1 or anti-Ubiquitin antibody in IP buffer overnight at 4 °C. Protein A-sepharose beads were added to the samples, which incubated for another 12 h. The sample were then washed and resuspended, and Western blotting was performed as described previously. The experiment was repeated three times.

### H&E, Masson’s, immunohistochemistry, immunofluorescence, TUNEL, and electron microscopy analysis

For histology, kidney tissues were fixed with 4% paraformaldehyde for paraffin embedding and hematoxylin & eosin staining. Masson’s trichrome staining was used to further evaluate renal fibrosis. Morphological changes in the kidney sections were assessed using immunohistochemistry (IHC), immunofluorescence, and transmission electron microscopy (TEM). Paraffin-embedded kidney sections used for IHC studies were dewaxed, rehydrated, and incubated with BiP (1:200) and LC3B (1:200) primary antibodies overnight at 4 °C. The sections were subsequently incubated with secondary antibodies, treated with diaminobenzidine, counterstained with hematoxylin and examined as previously reported. Immunofluorescence was performed by co-staining VDAC1 (1:200) and Ubiquitin (1:200) antibodies in the human kidney tubular epithelial cells. HK2 cells were stained using LAMP1 (1:200), Tomm20 (1:400), VDAC1 (1:200), P62 (1:200) and Ubiquitin (1:200) antibodies and secondary antibodies conjugated with Alexa Fluor. These cells were counterstained with DAPI, and their fluorescent signals were visualized using fluorescence microscope. The colocalization intensity of VDAC1, P62 or Ubiquitin was analyzed using Image J software.

For electron microscopy, fresh kidney tissues were fixed in a retrograde manner in 1.25% glutaraldehyde and 2% paraformaldehyde in a 0.1 M phosphate buffer and postfixed in 1% OsO_4_ in a 0.1 M phosphate buffer. Ultrathin sections (60 nm) were then cut on a microtome, placed on copper grids, stained with uranyl acetate and lead citrate, and examined under a transmission electron microscope (Tecnai G2 Spirit Twin, Holland).

For terminal deoxynucleotidyl transferase-mediated digoxigenindeoxyuridine nick-end labeling (TUNEL) staining, paraffin-embedded kidney tissue sections were stained with cell death detection kit (Beyotime, Shanghai, China). The slides were examined with fluorescent microscopy.

### Quantitative RT-PCR

Total RNA was extracted using TRIzol reagent and then reverse transcribed with Reverse Transcriptase (AG bio). Resulting complementary DNAs were quantified by real-time PCR using SYBR green master mix (AG bio) on the Step One Plus system (Applied Biosystems). The sequences of the primers were listed in supplementary materials (I).

### Statistical analysis

Results are presented as the means ± SEM. Data were analyzed by one-way ANOVA and Newman–Keuls tests for multiple comparisons. Statistical significance was accepted at the *P* < 0.05 level. The data analysis showed that the variance was homogeneous and accorded with normal distribution. Based on our preliminary experiments, sample sizes of all experiments were calculated in order to achieve a 90% power, with a 0.05 significance level.

## Supplementary information

supplementary data

FigureS1

FigureS2

FigureS3

FigureS4

FigureS5

FigureS6

FigureS7

FigureS8
