# Supplementary material for: Tubular Mas receptor mediates lipid-induced kidney injury
Source: Cell Death Dis. 2021 Jan 21;12(1):110. doi: 10.1038/s41419-020-03375-z (PMC7817966; doi:10.1038/s41419-020-03375-z)
Supplement: Supplementary file 1 — supplementary data [file 41419_2020_3375_MOESM1_ESM.docx]

**SUPPLEMENTARY MATERIALS:**

**Supplementary data I**

**Methods and Materials**

***Quantitative RT-PCR***

The sequences of the primers used were follows:

Mas1-F, 5’-AGAAATCCCTTCACGGTCTACA-3’；

Mas1-R, 5’-TCACCGATAATGTCACGATTGTG-3’;

VDAC1-F, 5’-ACGTATGCCGATCTTGGCAAA-3’;

VDAC1-R, 5’-TCAGGCCGTACTCAGTCCATC-3’;

LC3B-F, 5’-GTCTACGTGGGCCTTTACGC-3’;

LC3B-R, 5’-ACTTTCTTCCTAGCCTCCTTGTC-3’;

GRP78-F, 5’-ACTTGGGGACCACCTATTCCT-3’;

GRP78-R, 5’-GTTGCCCTGATCGTTGGCTA-3’;

DRP1-F, 5’- CCTCAGATCGTCGTAGTGGGA-3’;

DRP1-R, 5’- GTTCCTCTGGGAAGAAGGTCC -3’;

ACP2-F, 5’- GCATGTGCCTAACGGTGATG-3’;

ACP2-R, 5’- GTCCTTGGGATATGTCTTCACTG-3’;

UVRAG-F, 5’- ACATCGCTGCTCGGAACATT-3’;

UVRAG-R, 5’- CTCCACGTCGGATTCAAGGAA-3’;

CTSB-F, 5’- CAGGCTGGACGCAACTTCTAC-3’;

CTSB-R, 5’- TCACCGAACGCAACCCTTC-3’;

**Supplementary data II**

**Figure S1. (A)** and **(B)** Representative immunoblots and corresponding densitometry analysis of DRP1 protein abundance in the kidney cortex of Mas^+/+^ CTL, Mas^+/+^ HFD, Mas^-/-^ CTL and Mas^-/-^ HFD mice. **(C)** mRNA level of DRP1 in the kidney cortex of Mas^+/+^ CTL, Mas^+/+^ HFD, Mas^-/-^ CTL and Mas^-/-^ HFD mice. **(D)** Graphical representations of the degree of kidney fibrotic lesions in Mas^+/+^ CTL, Mas^+/+^ HFD, Mas^-/-^ CTL and Mas^-/-^ HFD mice after quantitative determination of Masson’s staining intensity. **(E)** The percentage of cells with TUNEL staining was evaluated. CTL, controls; HFD, high-fat diet. Data are shown as mean ± SEM; * P<0.05 compared with Mas^+/+^ CTL; # P<0.05 compared with Mas^+/+^ HFD. The experiment was repeated three times.

**Figure S2.** **(A)** and **(B)** Time-dependent changes in protein abundance of autophagy (P62 and LC3B), ER stress (BiP), and apoptosis (cleave caspase-3) markers in cultured PTCs prepared from Mas+/+ and Mas-/- mice after PA treatment. PTCs were treated with PA (400μM) for 1 hour, 3 hours, 6 hours, 12 hours, and 24 hours. β-actin was used as a loading control. (C) Confocal microscopy images of Mito-tracker (green) in PA-treated PTCs (12 hours and 24 hours) prepared from Mas+/+ and Mas-/- mice. CTL, controls; HFD, high-fat diet. Data are shown as mean ± SEM; * P<0.05 compared with Mas^+/+^ CTL; # P<0.05 compared with Mas^+/+^ HFD. The experiment was repeated three times.

**Figure S3. (A)** and **(B)** Representative immunoblots and corresponding densitometry analysis of DRP1 protein abundance in the kidney cortex of CTL, HFD, and HFD+A779 mice. **(C)** mRNA level of DRP1 in the kidney cortex CTL, HFD, and HFD+A779 mice. **(D)** Graphical representations of the degree of kidney fibrotic lesions in CTL, HFD and HFD+A779 mice after quantitative determination of Masson’s staining intensity. **(E)** The percentage of cells with TUNEL staining was evaluated.CTL, controls; HFD, high-fat diet; HFD+A779, high-fat diet plus A779 treatment. Data are shown as mean ± SEM; * P<0.05 compared with CTL; # P<0.05 compared with HFD. The experiment was repeated three times.

**Figure S4.** Palmitic acid (PA) induced autophagy stagnation and ER stress in HK2 cells. **(A)** Dynamic changes of LC3B and BiP protein expression in HK2 cells pretreated with 3-Methyladenine (3-MA) followed by PA overload. **(B)** 3-MA markedly inhibited protein abundance of LC3B and BiP in HK2 cells at the 24^th^ hour after PA treatment. **(C)** Dynamic changes of LC3B and BiP protein expression in HK2 cells pretreated with chloroquine (CQ) followed by PA overload. **(D)** CQ failed to relieve increased protein abundance of LC3B and BiP in HK2 cells at the 24^th^ hour after PA treatment. **(E)** Dynamic changes of LC3B and BiP protein expression in HK2 cells pretreated with rapamycin (RAPA) followed by PA overload. **(F)** RAPA markedly aggravated increased protein abundance of LC3B and BiP in HK2 cells at the 24^th^ hour after PA treatment. **(G)** Time-course images of LC3B puncta formation in HK2 cells transfected with LC3B -GFP-mCHERRY adenovirus pretreated with or without 3-MA, CQ or RAPA, followed by PA overload. **(H)** Quantification of total LC3 puncta in PA-treated HK2 cells with or without 3-MA, CQ or RAPA pretreatment. **(I)** The number of total LC3 puncta in HK2 cells after PA treated for 6 hours. **(J)** the ratio of yellow puncta (GFP and mCherry double positive) to all LC3B puncta (red) in HK2 cells after PA treated for 6 hours. In **A**, **C**, **E**, and **G**, HK2 cells were pretreated with 3-MA, CQ, or RAPA followed by PA overload for 3 hours, 6 hours, 12 hours and 24 hours, respectively. 3-MA, 3-Methyladenine; CQ, chloroquine; RAPA, rapamycin. Data are shown as mean ± SEM; * P<0.05 compared with CTL; # P<0.05 compared with PA. The experiment was repeated three times.

**Figure S5.** Dynamic changes of LC3B and BiP protein expression in HK2 cells pretreated with TUDCA followed by PA overload. TUDCA didn’t affect LC3B protein expression, but markedly suppressed unregulation of BiP protein in HK2 cells at the 24th hour after PA treatment. TUDCA, Tauroursedeoxycholic acid. Data are shown as mean SEM; * P<0.05 compared with CTL; # P<0.05 compared with PA. The experiment was repeated three times.

**Figure S6.** Sequencecing results of gRNA in Mas KD HK2 cells.

**Figure S7.** mRNA levels of ACP2, UVRAG, and CTSB were examined by quantitative RT-PCR in WT and Mas knockdown HK2 cells treated with PA (400μM) for 24h. Data are shown as mean ± SEM; * P<0.05 compared with WT CTL; The experiment was repeated three times.

**Figure S8.** Mas activation induced increases of intracellular calcium levels in HK2 cells treated with PA. **(A)** and **(B)** WT and Mas knockdown (KD) HK2 cells treated with PA (400μM) for 24 hours were immunostained with FLUO-4 AM probe and subjected to immunofluoresence or flow cytometric analysis. **(C)** Effect of AVE0991 or A779 on intracellular calcium level in HK2 cells treated with PA, assessed by FLUO-4AM analysis. WT, wildtype; KD, knockdown. Data are shown as mean ± SEM; * P<0.05 compared with WT CTL; # P<0.05 compared with WT PA. The experiment was repeated three times.
