# Supplementary figures and images for: Tubular Mas receptor mediates lipid-induced kidney injury
Source: Cell Death Dis. 2021 Jan 21;12(1):110. doi: 10.1038/s41419-020-03375-z (PMC7817966; doi:10.1038/s41419-020-03375-z)

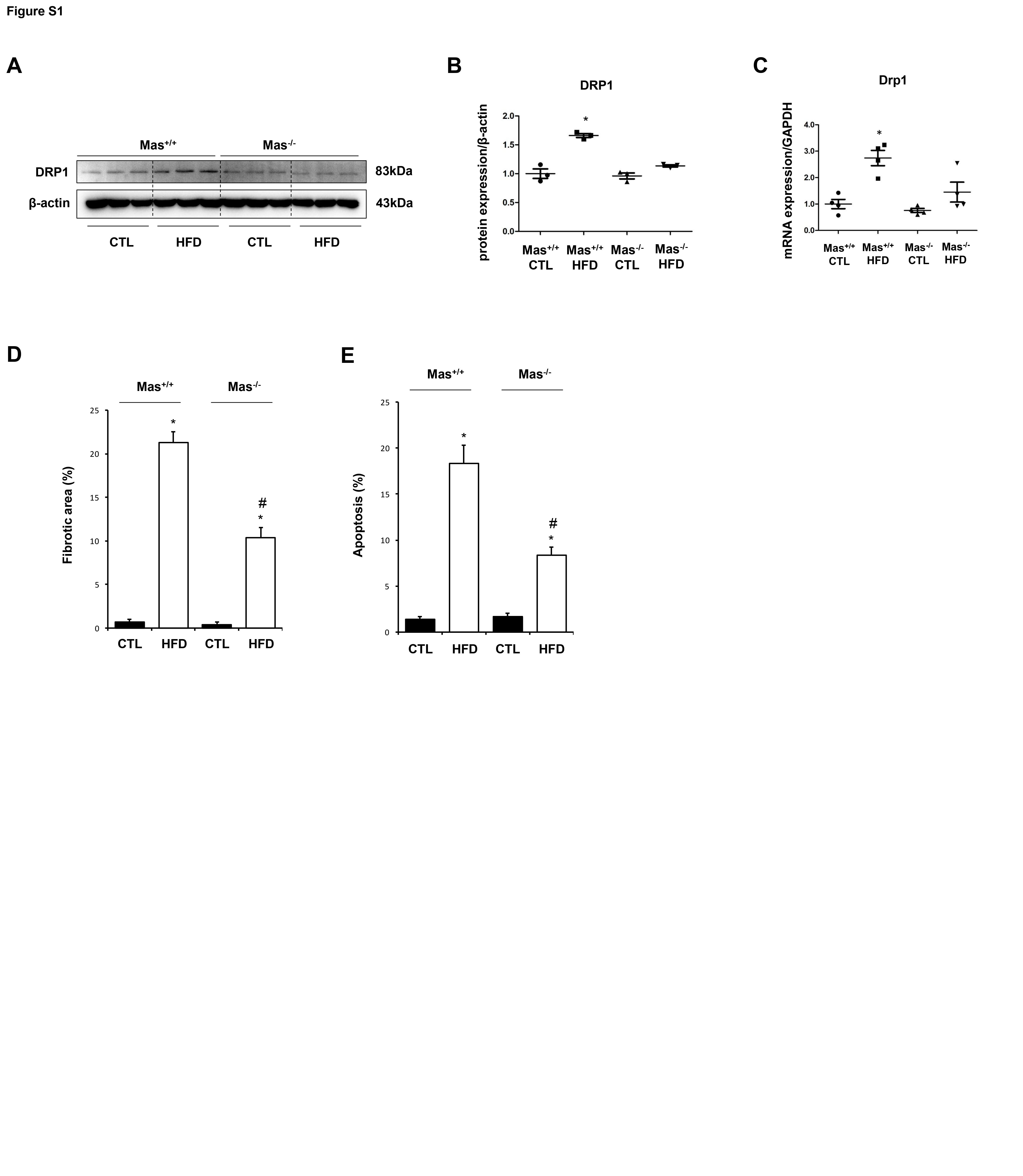

Supplement: Supplementary file 2 — FigureS1 [file 41419_2020_3375_MOESM2_ESM.png]

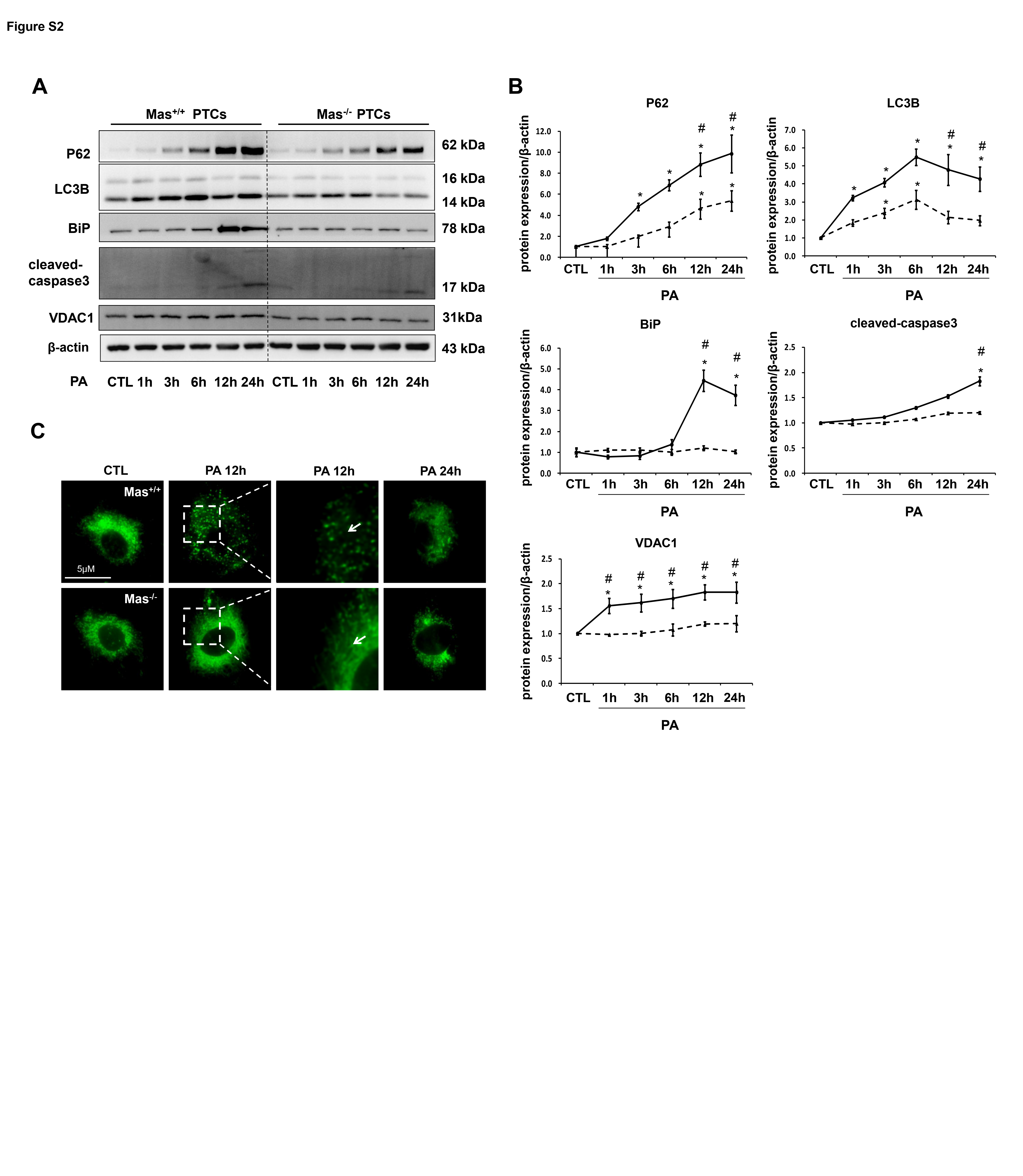

Supplement: Supplementary file 3 — FigureS2 [file 41419_2020_3375_MOESM3_ESM.png]

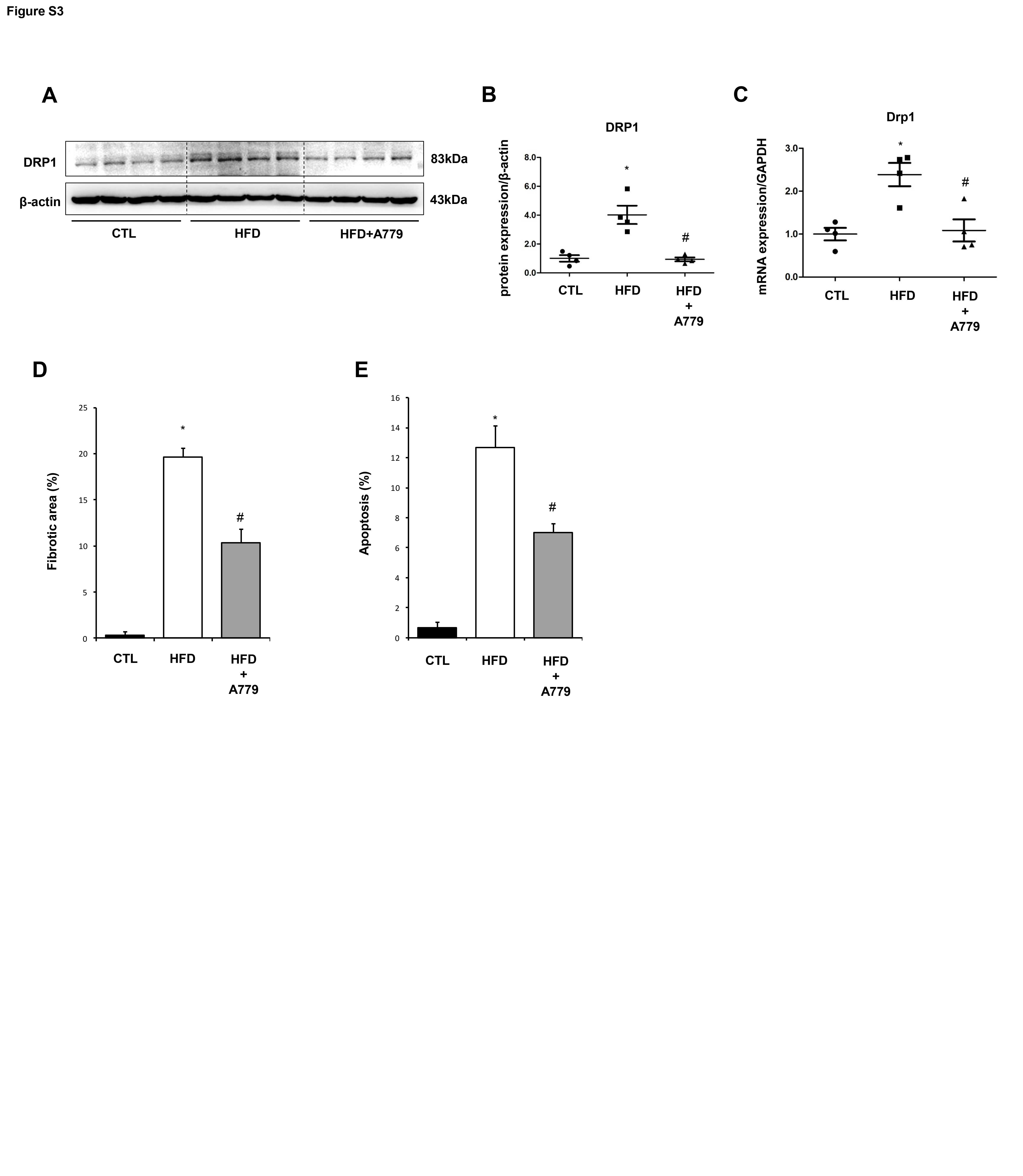

Supplement: Supplementary file 4 — FigureS3 [file 41419_2020_3375_MOESM4_ESM.png]

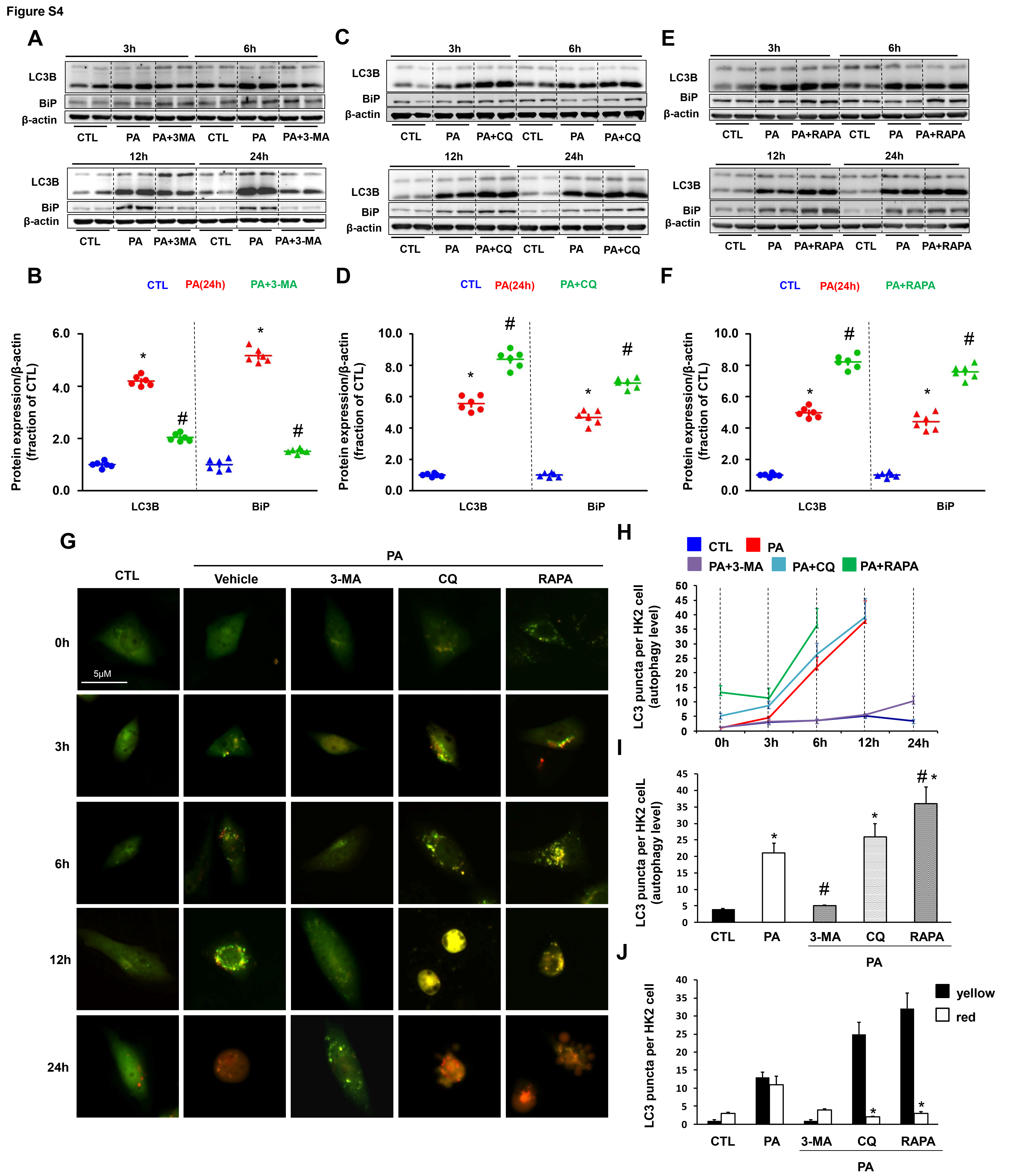

Supplement: Supplementary file 5 — FigureS4 [file 41419_2020_3375_MOESM5_ESM.png]

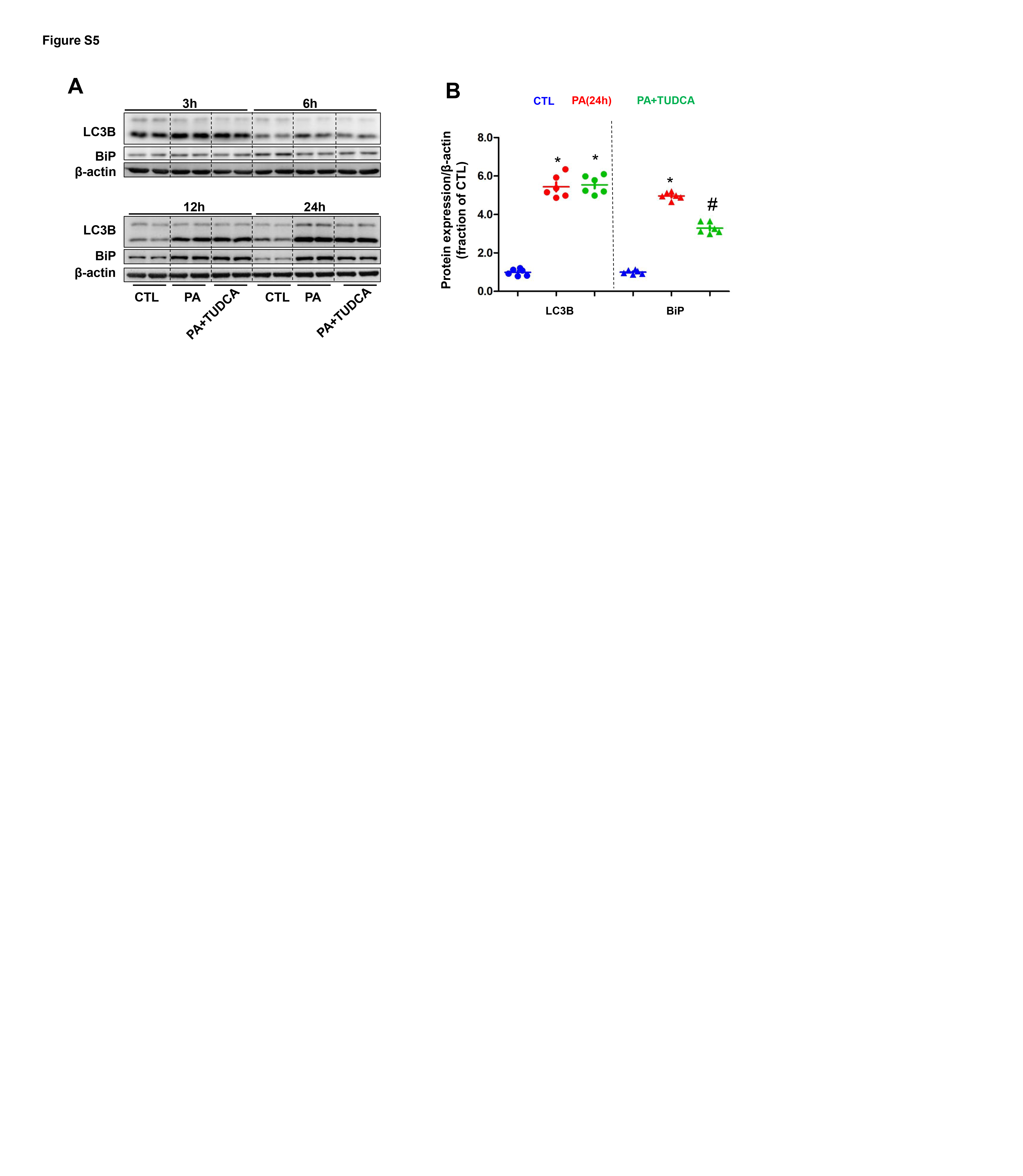

Supplement: Supplementary file 6 — FigureS5 [file 41419_2020_3375_MOESM6_ESM.png]

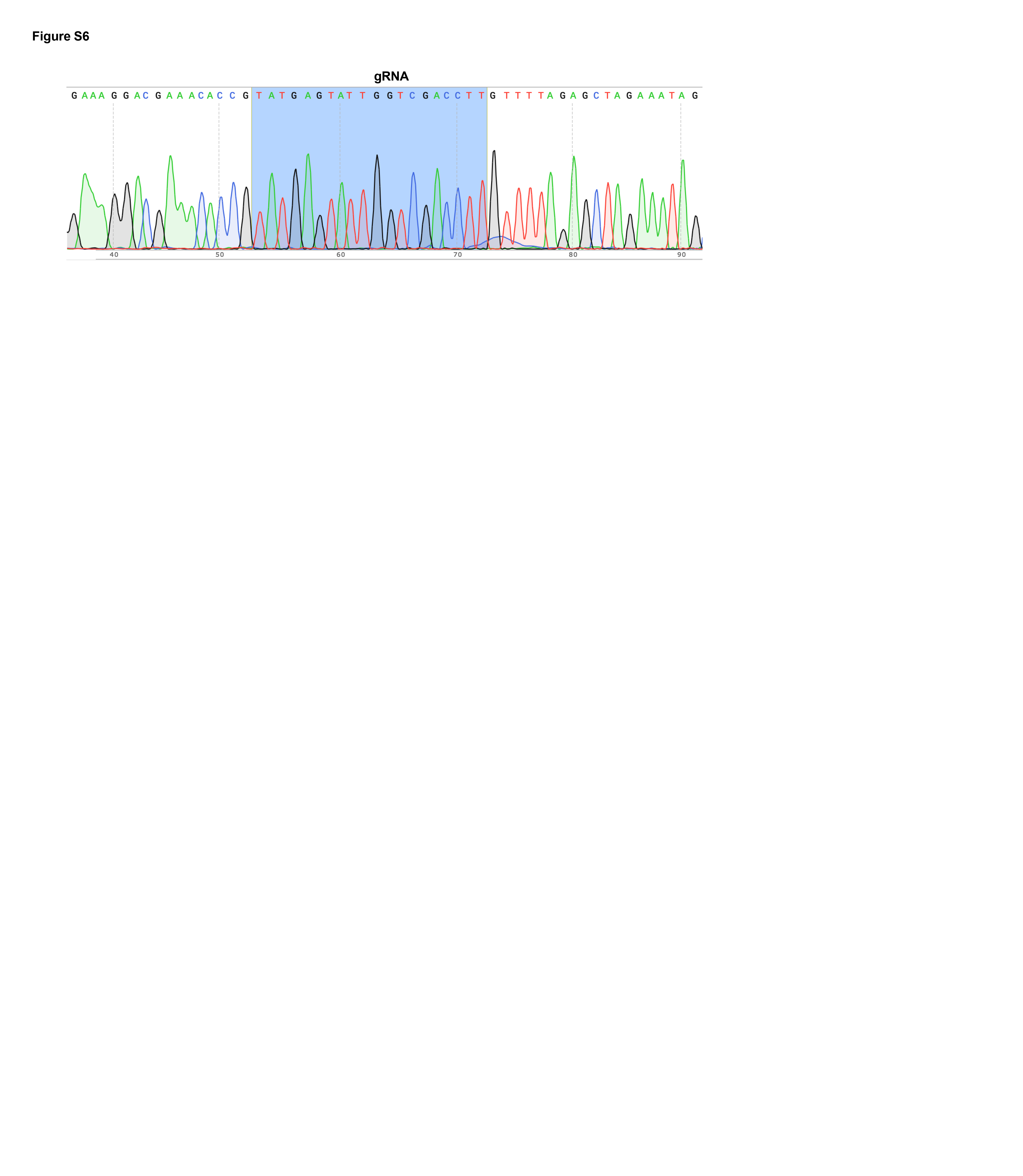

Supplement: Supplementary file 7 — FigureS6 [file 41419_2020_3375_MOESM7_ESM.png]

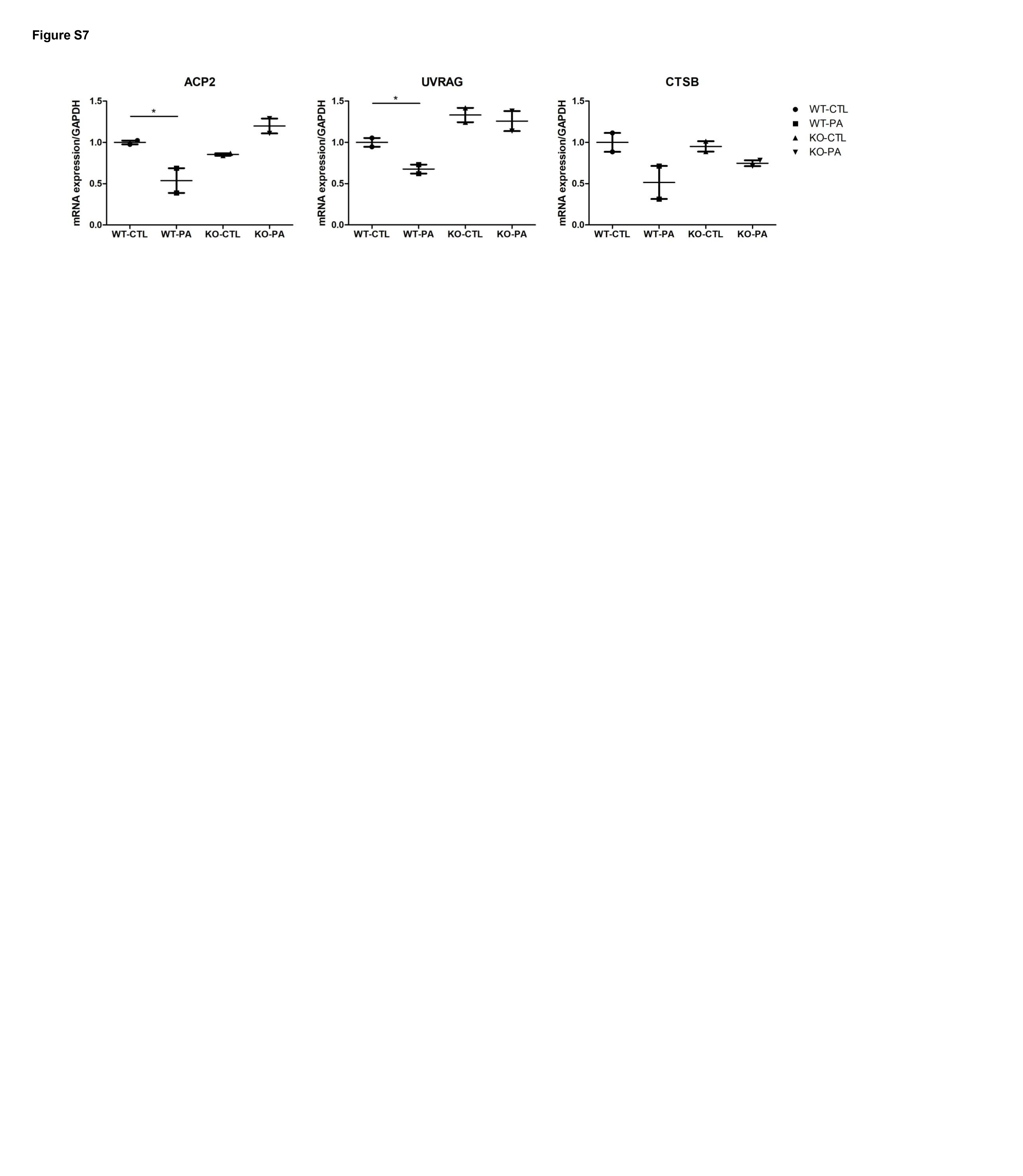

Supplement: Supplementary file 8 — FigureS7 [file 41419_2020_3375_MOESM8_ESM.png]

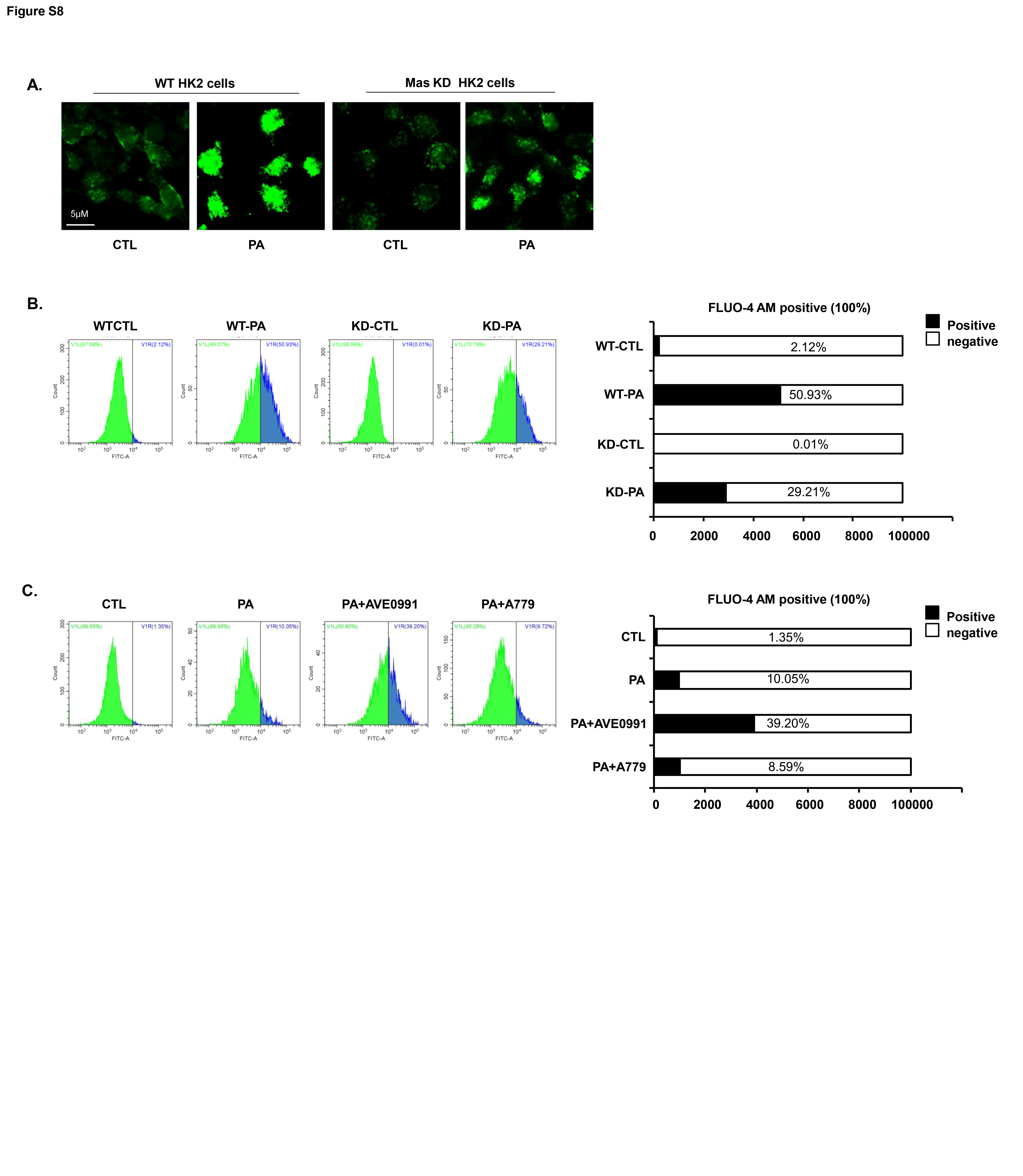

Supplement: Supplementary file 9 — FigureS8 [file 41419_2020_3375_MOESM9_ESM.png]
